# An Efficient Supervised Training Algorithm for Multilayer Spiking Neural Networks

**DOI:** 10.1371/journal.pone.0150329

**Published:** 2016-04-04

**Authors:** Xiurui Xie, Hong Qu, Guisong Liu, Malu Zhang, Jürgen Kurths

**Affiliations:** 1 Department of Computer Science and Engineering, University of Electronic Science and Technology of China, 611731, Chengdu, Sichuan, China; 2 Department of Physics, Humboldt University, 12489, Berlin, Berlin, Germany; 3 Potsdam Institute for Climate Impact Research(PIK), 14473 Potsdam, Germany; Georgia State University, UNITED STATES

## Abstract

The spiking neural networks (SNNs) are the third generation of neural networks and perform remarkably well in cognitive tasks such as pattern recognition. The spike emitting and information processing mechanisms found in biological cognitive systems motivate the application of the hierarchical structure and temporal encoding mechanism in spiking neural networks, which have exhibited strong computational capability. However, the hierarchical structure and temporal encoding approach require neurons to process information serially in space and time respectively, which reduce the training efficiency significantly. For training the hierarchical SNNs, most existing methods are based on the traditional back-propagation algorithm, inheriting its drawbacks of the gradient diffusion and the sensitivity on parameters. To keep the powerful computation capability of the hierarchical structure and temporal encoding mechanism, but to overcome the low efficiency of the existing algorithms, a new training algorithm, the Normalized Spiking Error Back Propagation (NSEBP) is proposed in this paper. In the feedforward calculation, the output spike times are calculated by solving the quadratic function in the spike response model instead of detecting postsynaptic voltage states at all time points in traditional algorithms. Besides, in the feedback weight modification, the computational error is propagated to previous layers by the presynaptic spike jitter instead of the gradient decent rule, which realizes the layer-wised training. Furthermore, our algorithm investigates the mathematical relation between the weight variation and voltage error change, which makes the normalization in the weight modification applicable. Adopting these strategies, our algorithm outperforms the traditional SNN multi-layer algorithms in terms of learning efficiency and parameter sensitivity, that are also demonstrated by the comprehensive experimental results in this paper.

## Introduction

Increasing the level of realism in a neural simulation and improving the computational capability of artificial neural networks [1][2], the spiking neural networks employing temporal coding mechanism is introduced as the third generation of neural networks and has achieved great success in various artificial intelligence tasks [3]–[6]. Most traditional neural networks represent real-valued analog data by the firing rate of neurons, like the first generation neural networks pioneered by the McCulloch-Pitts model [7] and the second generation by the the perceptron model [8]. However, there are substantial evidences that in biological neural systems there exist fast computations that are very likely based on spike firing events [1][2][9]. To simulate these firing events, the third generation of neural networks, the SNNs transmit information by spike times instead of the firing rate, and have been proven computationally more powerful than networks with rate coding [10]–[13].

In the learning of SNNs with temporal encoding mechanism, the supervised training is an important biomimetic concept which could potentially improve the learning speed with the help of an instructor signal. Various supervised training algorithms of SNNs have been proposed by now, which can broadly be subdivided into two classes: training algorithms for single layer SNNs, and for multilayers.

The single layer training algorithms are introduced based on the gradient decent rule or learning windows. Regarding to the gradient decent rule, the Tempotron [14] is a classical algorithm employing the distance between the output neuron’s voltage and the firing threshold as the cost function and can complete training efficiently, however, it can only complete binary classification tasks. The Chronotron [15] and spike pattern association neuron algorithm [16] try to minimize the distance between the desired and actual output spike trains by the gradient descent rule, with the distance defined by the Victor and Purpura metric [17] and the van Rossum metric [18] respectively.

A lot of algorithms based on learning windows have been proposed [19] for single layer networks. Among which, the remote supervised learning method is a classical one employing both the Spike-Timing-Dependent Plasticity (STDP) window, and the anti-STDP learning window to complete training [20]. The perceptron-based spiking neuron learning rule adopts a learning window based on the postsynaptic voltage function to instruct training [21]. The Synaptic Weight Association Training (SWAT) utilizes the STDP learning window and the Bienenstock-Cooper-Munro learning rule [22] to drive learning and achieves convergence. The precise spike driven synaptic plasticity learning rule [23] combines the Windrow-Hoff rule and the learning window of postsynaptic potential. Further algorithms adopting learning windows are introduced in [24]. These training algorithms employing learning windows are often more efficient than those with the gradient descent rule. But these single layer algorithms cannot complete training when the network structure contains hidden layers. However, electrophysiology experiments on cat’s visual system and monkey striate cortex reveal that the information in biological neurons is processed hierarchically rather than by a single layer [25]–[27]. Then, training a hierarchical spiking neural network is by far the closest way to the biological system.

For multilayers learning of the SNNs, the Spike Propagation (SpikeProp) [28] is the pioneer method that defines the computational error by the distance between the actual and target firing time, and minimizes the error by the gradient descent rule. It achieves training accurately but inefficiently, and only the first spike of a neuron can be trained. Different variations of the SpikeProp, the Quick Propagation, Resilient Propagation [29] and the Multiple SpikeProp [30][31] are proposed to improve the SpikeProp’s learning performance. The Multi-layer Remote Supervised Learning Method(Multi-ReSuMe) [32] extends the ReSuMe [20] to multiple layers by the gradient decent rule, assuming that the relation between the input and output firing rates is linear. All of these existing algorithms can achieve learning, while the efficiency of them is much lower than that of the biological system [33][34], and does not meet the requirements of the real-time applications.

To solve the low efficiency problem in the multilayer training of SNNs, the Normalized Spiking Error Back-Propagation (NSEBP) is proposed in this paper, which is motivated by the selective attention mechanism of the primate visual system [35, 36] and its layer-wise feature representation method in hierarchical structures [25, 26]. Different from traditional algorithms, our algorithm only selects target spike times as attention areas and ignores the states of other times. Besides, the voltage difference is employed to evaluate training errors, and the relation between the weight variation and voltage error change is uncovered, which enables the NSEBP to adjust each synaptic efficacy accurately. Moreover, the computational error is back propagated to previous layers by presynaptic spike jitter instead of the traditional gradient decent rule, which realizes layer-wise training in our algorithm. In the feedforward calculation, the analytic solutions of the spike time are calculated in the spiking response model, instead of detecting the postsynaptic voltage states at all time points. Employing these strategies, our algorithm achieves a significant improvement in training efficiency compared with the traditional training methods.

## Learning Algorithm

In this section, a new algorithm for feed-froward multilayer spiking neural networks, the Normalized Spiking Error Back Propagation (NSEBP) is presented.

### Neuron Model

In our study, the simplified Spike Response Model (SRM_0_) is employed because of its simplicity and effectiveness. In the SRM_0_ [37], once the *j*th spike is emitted, a fundamental voltage *ϵ*_*j*_ is inspired and transmitted to its postsynaptic neuron. Each postsynaptic neuron integrates the weighted sum of all presynaptic influence *ϵ*_*j*_ at time *t* as its voltage *u*(*t*), and emits a spike if its voltage *u*(*t*) reaches the threshold. The postsynaptic voltage *u*(*t*) is described in the following equations:

$$\begin{array}{ccc} u(t) & = & \eta \left(t-{\hat{t}}_{out}\right)+\sum_{j\in \Gamma_{j}}w_{j}\epsilon_{j}\left(t-t_{in}^{j}\right)+u^{ext}, \end{array}$$ (1)

where

$$\begin{array}{c} \epsilon_{j}\left(s_{j}\right)=\left[\exp\left(-\frac{s_{j}}{\tau_{1}}\right)-\exp\left(-\frac{s_{j}}{\tau_{2}}\right)\right]H\left(s_{j}\right), \end{array}$$ (2)

with the Heaviside step function

$$\begin{array}{ccc} H(s_{j}) & = & \left\{ \begin{array}{cc} 1, & \text{if}\;\;s_{j}\geq 0,\\ 0, & \text{otherwise}. \end{array}\right. \end{array}$$ (3)

Specifically, ${\hat{t}}_{out}$ denotes the last recent output spike of the postsynaptic neuron, *w*_*j*_ is the weight of the presynaptic neuron emitting the *j*th input spike, and $\eta (t-{\hat{t}}_{out})$ is the refractory function to simulate the biological refractory period. Γ_*j*_ is a set containing the spike time emitted by all the presynaptic neurons, *u*^*ext*^ is the external voltage, $s_{j}=t-t_{in}^{j}$, with $t_{in}^{j}$ denoting the *j*th firing time of the input spike train. *τ*_1_ and *τ*_2_ are constant parameters.

In our algorithm, the Post-Synaptic Potential (PSP) learning window is employed, which is represented in Eq (4), providing a relation of the weight modification and spike time deviation. Obviously, it only directs weight modification if the presynaptic neuron fires before the postsynaptic one.

$$\begin{array}{ccc} W_{ind}\left(s_{j}\right) & = & \left\{ \begin{array}{cc} A_{1}\epsilon_{j}\left(s_{j}\right), & \text{if}\;\;s_{j}\geq 0,\\ 0, & \text{otherwise}. \end{array}\right. \end{array}$$ (4)

where *A*_1_ is a constant set to be 1 in our study, and $s_{j}=t-t_{in}^{j}$ denotes the time distance between the current time *t* and the input time $t_{in}^{j}$.

### The NSEBP Algorithm

In this section, the Normalized Spiking Error Back Propagation (NSEBP) is proposed. The feedforward calculation process is derived in the Theorem 1, and the feedback training process of this learning rule is described here for one postsynaptic neuron with several presynaptic neurons.

In the network with *n* layers employing the SRM_0_ model, an arbitrary postsynaptic neuron *o* has an input spike train $T_{in}=\left\{t_{in}^{1},t_{in}^{2},t_{in}^{3},\ldots t_{in}^{P}\right\}$ denoting the ordered input spikes, and a target spike train $T_{d}=\left\{t_{d}^{1},t_{d}^{2},\ldots ,t_{d}^{D}\right\}$. Inspired by the selective attention mechanism of the primate visual system, the NSEBP only detects and trains the voltage states at target time points for neuron *o*, and ignore states on other non-target time.

For each postsynaptic neuron *o*, instead of the traditional time error, the voltage distance between the threshold *ϑ* and the postsynaptic voltage *u*(*t*_*d*_) at the target time *t*_*d*_ is employed as the network error in our algorithm described in Eq (5), which is trained to become zero in our algorithm:

$$\begin{array}{c} \text{err}=\vartheta -u(t_{d}) \end{array}$$ (5)

To train the postsynaptic voltage to *ϑ*, two steps are applied to our algorithm, that are the presynaptic spike jitter to back propagate error and the weight modification to complete training of the current layer.

#### Presynaptic spike jitter

**Fig 1:**
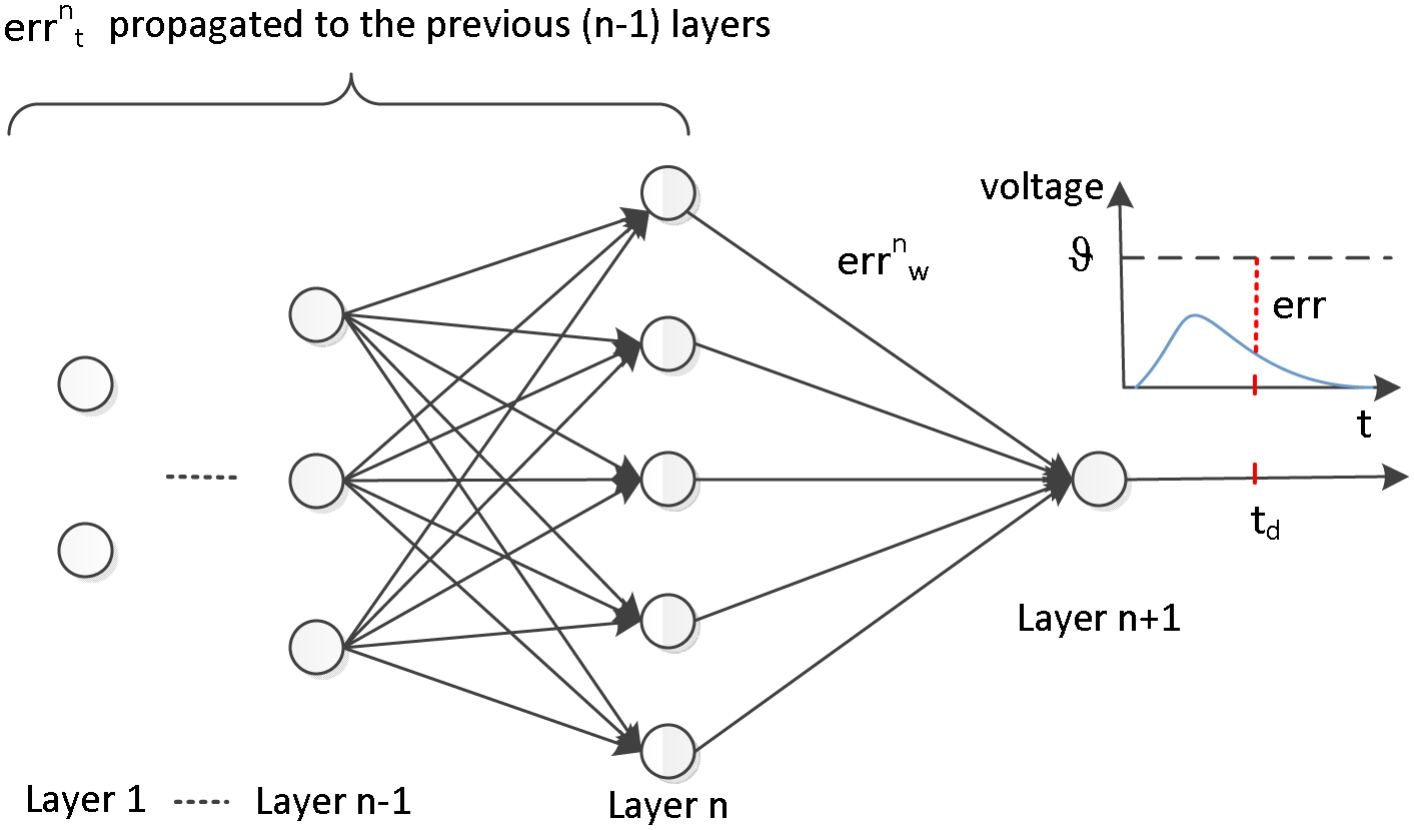
The error and its assignment in our algorithm. The error *err* is assigned to two parts, among which $err_{w}^{n}$ is assigned to the current layer for weight modification, and $err_{t}^{n}$ is propagated to previous (n − 1) layers.

The presynaptic spike jitter is employed to back propagate error instead of the traditional gradient descent rule. It can influence the postsynaptic neuron voltage *u*(*t*_*d*_) and realize layer-wised training. To achieve the back-propagated layer-wised learning, neurons in hidden layers also require the target spike time and training error. Then, the error in Eq (5) is allocated to *n* layers by the normalized parameter *r* and back propagated by the presynaptic spike jitter. The error assigned to the current layer $err_{w}^{n}$ is

$$\begin{array}{c} err_{w}^{n}=r\text{err}, \end{array}$$ (6)

and to the previous *n* − 1 layers $err_{t}^{n}$ is

$$\begin{array}{c} err_{t}^{n}=\left(1-r\right)\text{err}, \end{array}$$ (7)

where *r* is set to 1/*n* in our algorithm. This error assignment is shown in Fig 1.

The error $err_{t}^{n}$ is back propagated to previous *n* − 1 layers by shifting each influential presynaptic spikes (presynaptic spikes which have influence to the postsynaptic neuron *o* at the current target time *t*_*d*_). The error assigned to the *j*th influential presynaptic spike is Δ*u*_*j*_, which is calculated by

$$\begin{array}{c} \Delta u_{j}=\gamma_{t}^{jn}err_{t}^{n}. \end{array}$$ (8)

In which $\gamma_{t}^{jn}$ is an assign variable and calculated by

$$\begin{array}{ccc} \gamma_{t}^{jn} & = & \left\{ \begin{array}{cc} \left(A_{1}-W_{ind}\left(s_{j}\right)\right)/\sum_{k=m_{1}}^{m_{2}}\left(A_{1}-W_{ind}\left(s_{k}\right)\right), & \text{if}\;\;\text{err}>0,\\ W_{ind}\left(s_{j}\right)/\sum_{k=m_{1}}^{m_{2}}W_{ind}\left(s_{k}\right), & \text{if}\;\;err<0, \end{array}\right. \end{array}$$ (9)

with *A*_1_ and *W*_*ind*_(*s*_*j*_) defined in Eq (4). *m*_1_ and *m*_2_ are the first and last indexes of the influential presynaptic spikes respectively. To achieve training of this Δ*u*_*j*_, the time variation of the *j*th input spike $\Delta t_{pre}^{j}$ is calculated by

$$\begin{array}{c} \Delta t_{pre}^{j}=\tau_{1}\ln\left(\frac{-b\pm \sqrt{b^{2}-4ac}}{2a}\right), \end{array}$$ (10)

with

$$\begin{array}{ccc} a & = & -w_{j}\exp\left(\left(t_{pre}^{j}-t_{d}\right)/\tau_{2}\right), \end{array}$$ (11)

$$\begin{array}{ccc} b & = & w_{j}\exp\left(\left(t_{pre}^{j}-t_{d}\right)/\tau_{1}\right), \end{array}$$ (12)

$$\begin{array}{ccc} c & = & w_{j}\exp\left(\left(t_{pre}^{j}-t_{d}\right)/\tau_{2}\right)-w_{j}\exp\left(\left(t_{pre}^{j}-t_{d}\right)/\tau_{1}\right)-\Delta u_{j}, \end{array}$$ (13)

where *w*_*j*_ is the corresponding weight of the *j*th presynaptic spike, *t*_*d*_ is the target spike time, *τ*_1_ and *τ*_2_ are model parameters defined in Eq (2), $t_{pre}^{j}$ denotes the *j*th presynaptic spike time. If *a* ≠ 0 and *b*^2^ − 4*ac* ≥ 0 hold, $\Delta t_{pre}^{j}$ is calculated by Eq (10) and the solution with the minimum absolute value is applied to our algorithm. The calculation is derived in the following theoretical derivation section.

#### Weight modification

To train $err_{w}^{n}$ to 0, the weight modification in our algorithm for an arbitrary influential input spike *j* is calculated by

$$\begin{array}{c} \Delta w_{j}=\frac{\gamma_{j}^{n}err_{w}^{n}}{\epsilon_{j}\left(s_{j}\right)}, \end{array}$$ (14)

where $\gamma_{j}^{n}$ is a parameter defined by the normalized learning window

$$\begin{array}{c} \gamma_{j}^{n}=\frac{W_{ind}\left(s_{j}\right)}{\sum_{k=m_{1}}^{m_{2}}W_{ind}\left(s_{k}\right)}, \end{array}$$ (15)

with *ϵ*_*j*_(*s*_*j*_) calculated by Eq (2), and *W*_*ind*_(*s*_*j*_) by Eq (4).

**Fig 2:**
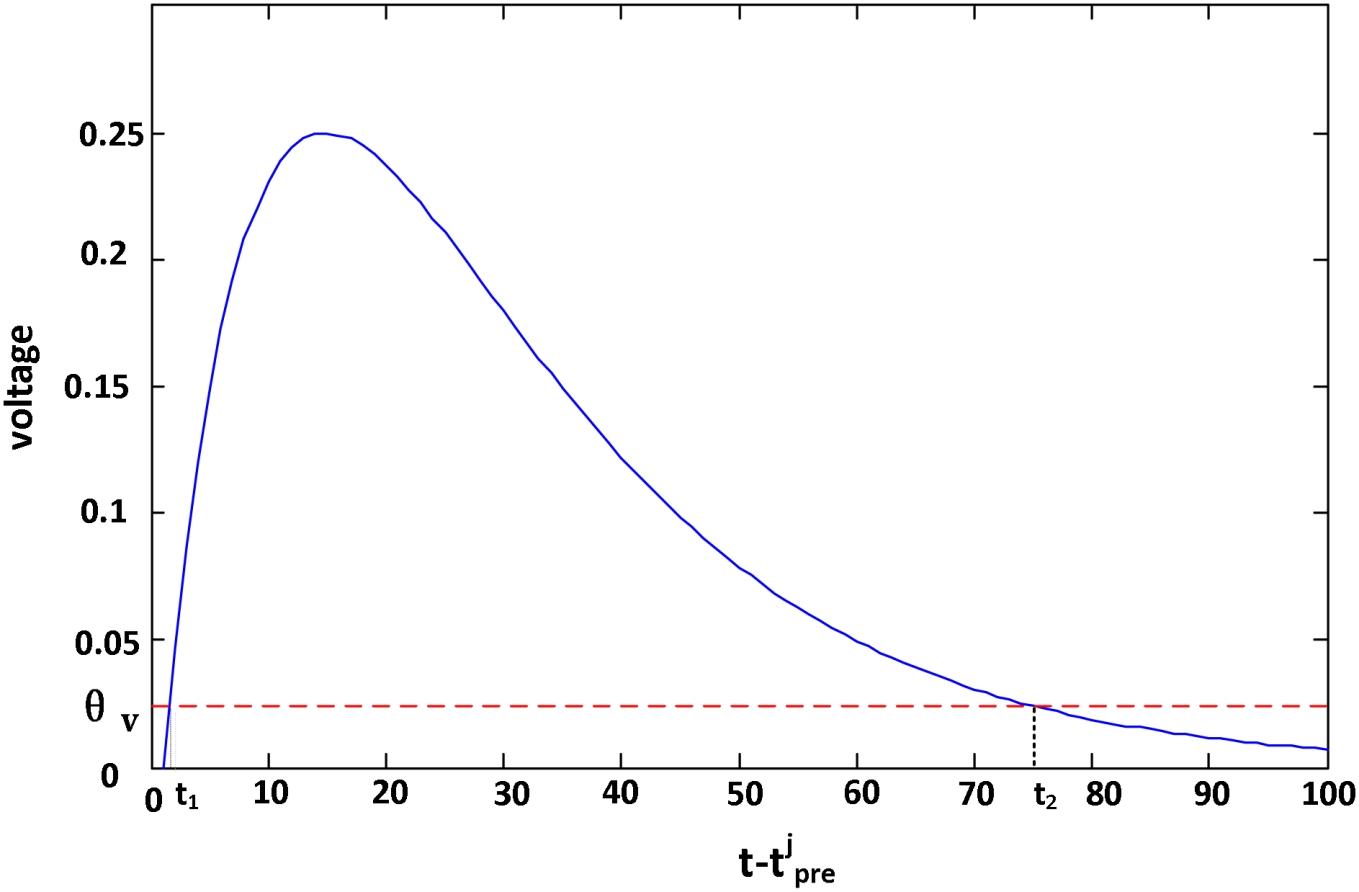
The voltage in the time scope $t-t_{pre}^{j}\in \left[t_{1},t_{2}\right]$. The voltage *ϵ*_*j*_(*s*_*j*_) caused by the input spike $t_{pre}^{j}$ is above *ϑ*_*v*_ when $t-t_{pre}^{j}\in \left[t_{1},t_{2}\right]$. The voltage of the input $t_{pre}^{j}$ is set to 0 at time *t* if this $t-t_{pre}^{j}$ is not in the interval [*t*_1_, *t*_2_].

To avoid the *ϵ*_*j*_(*s*_*j*_) in Eq (14) going to infinitesimal when *s*_*j*_ is too large, not all presynaptic spikes but only the spikes with voltage *ϵ*_*j*_(*s*_*j*_) > *ϑ*_*v*_ at *t*_*d*_ are trained, where *ϑ*_*v*_ is the voltage threshold. Solving the same mathematical equations as Theorem 2 in the following section, the time boundaries are $t_{1}=t_{d}-t_{pre}^{j}+\tau_{1}\ln\left(\left(1-\sqrt{1-4\vartheta_{v}}\right)/2\right)$ and $t_{2}=t_{d}-t_{pre}^{j}+\tau_{1}\ln\left(\left(1+\sqrt{1-4\vartheta_{v}}\right)/2\right)$, as shown in Fig 2. Its corresponding spike index range is denoted by [*m*_1_, *m*_2_].

If there is no input spike in the range [*t*_1_, *t*_2_], *S* spikes in [*t*_1_, *t*_2_] are added randomly to the presynaptic hidden neurons with a probability. The probability *p*_*i*_ assigned spikes to neuron *i* is calculated by

$$\begin{array}{c} p_{i}=\frac{\frac{1}{n_{i}}}{\sum_{k=1}^{m}\frac{1}{n_{k}}}, \end{array}$$ (16)

in which, *m* is the number of presynaptic neurons, *n*_*i*_ is the number of spikes emitted by neuron *i*, and *n*_*i*_ = 0.5 if there is no spike emitted. Consequently, the fewer spikes emitted by neuron *i*, the higher probability *p*_*i*_ it possesses. This allocation approach not only solves the none input problem in the training, but also balances the spike distribution. These added spikes are regarded as the target time of previous hidden layers and trained in the previous layer.

## Mathematical Analysis

### Theoretical Derivation

In this section, the theoretical derivations in the feedforward and back propagation of our algorithm are presented in Theorem 1 and Theorem 2 respectively.

In traditional methods employing the temporal encoded SNNs, the output spikes of a neuron are detected serially in time, leading to an inefficient feed forward process. In our study, the output spike times are obtained by solving the voltage function instead of traversing all time points, which are derived in the following theorem.

Supposing that for a postsynaptic neuron *o*, the refractory period $\eta (t-{\hat{t}}_{out})$ is set to $-A_{2}\exp\left(-\left(t-{\hat{t}}_{out}\right)/\tau_{1}\right)$ with the constant *A*_2_ > 0, the external interference voltage *u*^*ext*^ = 0, and $T_{pre}=\{t_{pre}^{1},t_{pre}^{2},t_{pre}^{3},\ldots t_{pre}^{P_{1}}\}$ is the ordered presynaptic spike train of the *P*_1_ presynaptic input spikes, where $t_{pre}^{j}$ denotes the *j*th presynaptic spikes with *w*_*j*_ representing its response synapse weight. *m*_*o*_ is the index of the first influencing spike to the postsynaptic neuron *o*, *ϑ* is the firing threshold, *τ*_1_ and *τ*_2_ are model parameters defined in Eq (2). With these definitions, the relation between the pre and postsynaptic spikes is obtained by solving the quadratic function, which is proved in the following theorem:

**Theorem 1** *In the* *SRM*_0_ *model, for each range* $\left[t_{pre}^{j},t_{pre}^{j+1}\right)$ *in* *T*_*pre*_ *with* 1 ≤ *j* ≤ *P*_1_ − 1, *assuming that*

$$\begin{array}{ccc} a & = & \sum_{m=m_{0}}^{j}w_{m}\exp\left(t_{pre}^{m}/\tau_{2}\right), \end{array}$$ (17)

$$\begin{array}{ccc} b & = & \sum_{m=m_{0}}^{j}w_{m}\exp\left(t_{pre}^{m}/\tau_{1}\right)-A_{2}\exp\left({\hat{t}}_{out}/\tau_{1}\right) \end{array}$$ (18)

*If the following conditions hold*:

$$\begin{array}{ccc} \left(I\right) & & a\neq 0\;\text{and}\;b^{2}-4a\vartheta \geq 0,\\ \left(II\right) & & \tau_{1}=2\tau_{2}, \end{array}$$

*the postsynaptic output spike* *t*_*out*_ *in the range* $\left[t_{pre}^{j},t_{pre}^{j+1}\right)$ *is solved by*

$$\begin{array}{c} t_{out}=-\tau_{1}\ln(\frac{b\pm \sqrt{b^{2}-4a\vartheta }}{2a}). \end{array}$$ (19)

***Proof***: According to the SRM_0_ model described in Eqs (1)–(3), for *t* ∈ [$t_{pre}^{j},t_{pre}^{j+1}$), the voltage of a postsynaptic neuron *u*(*t*) is

$$\begin{array}{ccc} u(t) & = & \eta \left(t-{\hat{t}}_{out}\right)+\sum_{m=m_{0}}^{j}w_{m}\epsilon_{m}\left(t-t_{pre}^{m}\right)+u^{ext}. \end{array}$$ (20)

Since

$$\begin{array}{c} \left\{ \begin{array}{c} \eta \left(t-{\hat{t}}_{out}\right)=-A_{2}\exp\left(-\left(t-{\hat{t}}_{out}\right)/\tau_{1}\right),\\ u^{ext}=0, \end{array}\right. \end{array}$$ (21)

at time *t*_*out*_, we have

$$\begin{array}{c} u\left(t_{out}\right)=\sum_{m=m_{0}}^{j}w_{m}\epsilon_{m}\left(t_{out}-t_{pre}^{m}\right)-A_{2}\exp\left(-\frac{t_{out}-{\hat{t}}_{out}}{\tau_{1}}\right). \end{array}$$ (22)

The postsynaptic neuron will fire once its voltage reaches the threshold *ϑ*, then the postsynaptic output spike time *t*_*out*_ follows

$$\begin{array}{c} \sum_{m=m_{0}}^{j}w_{m}\epsilon_{m}\left(t_{out}-t_{pre}^{m}\right)-A_{2}\exp\left(-\frac{t_{out}-{\hat{t}}_{out}}{\tau_{1}}\right)=\vartheta . \end{array}$$ (23)

According to Eq (2),

$$\begin{array}{c} \sum_{m=m_{0}}^{j}w_{m}\left[\exp\left(-\frac{t_{out}-t_{pre}^{m}}{\tau_{1}}\right)-\exp\left(-\frac{t_{out}-t_{pre}^{m}}{\tau_{2}}\right)\right]-A_{2}\exp\left(-\frac{t_{out}-{\hat{t}}_{out}}{\tau_{1}}\right)=\vartheta . \end{array}$$ (24)

Thus, we have

$$\begin{array}{ccc} & & \sum_{m=m_{0}}^{j}w_{m}\left\{\exp\left(\frac{-t_{out}}{\tau_{1}}\right)\exp\left(\frac{t_{pre}^{m}}{\tau_{1}}\right)-\exp\left(\frac{-t_{out}}{\tau_{2}}\right)\exp\left(\frac{t_{pre}^{m}}{\tau_{2}}\right)\right\}-A_{2}\exp\left(\frac{-t_{out}}{\tau_{1}}\right)\exp\left(\frac{{\hat{t}}_{out}}{\tau_{1}}\right)\\ & & \;=\vartheta . \end{array}$$ (25)

Suppose

$$\begin{array}{c} z=\exp(-\frac{t_{out}}{\tau_{1}}), \end{array}$$

and refer to Eqs (17), (18), (25) and (*II*),

$$\begin{array}{c} az^{2}-bz+\vartheta =0. \end{array}$$ (26)

By (*I*), the solutions of Eq (26) is

$$\begin{array}{c} z=\frac{b\pm \sqrt{b^{2}-4a\vartheta }}{2a}, \end{array}$$ (27)

and for all presynaptic time, we have *t*_*i*_ > 0, *z* > 0, then

$$\begin{array}{c} t_{out}=-\tau_{1}\ln\left(\frac{b\pm \sqrt{b^{2}-4a\vartheta }}{2a}\right). \end{array}$$ (28)

The result follows.

The Theorem 1 proves the relation between the pre and postsynaptic spikes, which is applied to our algorithm to improve the feedforward computation efficiency.

In the feedback process of our algorithm, the error is back propagated by the presynaptic spike jitter instead of the traditional gradient decent rule, by which, the layer-wised training is applicable to our algorithm and improves the learning efficiency of our algorithm significantly. The relation of the presynaptic spike jitter and the voltage change is investigated in the following theorem.

Supposing that Δ*u*_*j*_ is the voltage variation of the postsynaptic neuron *o* generated by the *j*th presynaptic spike, *t*_*d*_ is the current target spike time, and other variables are the same as that in Theorem 1, then the relation between the time jitter $\Delta t_{pre}^{j}$ and Δ*u*_*j*_ is obtained by solving the quadratic function, which is proved in the following theorem:

**Theorem 2** *In the* *SRM*_0_ *model with* *τ*_1_ = 2*τ*_2_, *if the voltage change* Δ*u*_*j*_ *follows*

$$\begin{array}{ccc} \left(I\right) & & w_{j}\neq 0,\\ \left(II\right) & & -w_{j}\epsilon_{j}\left(t_{d}-t_{pre}^{j}\right)<\Delta u_{j}\leq \left(\frac{1}{4}-\epsilon_{j}\left(t_{d}-t_{pre}^{j}\right)\right)w_{j},\;\;\text{if}\;\;w_{j}>0\\ \left(III\right) & & \left(\frac{1}{4}-\epsilon_{j}\left(t_{d}-t_{pre}^{j}\right)\right)w_{j}\leq \Delta u_{j}<-w_{j}\epsilon_{j}\left(t_{d}-t_{pre}^{j}\right),\;\;\text{if}\;\;w_{j}<0 \end{array}$$

*and assuming that*

$$\begin{array}{ccc} a & = & -w_{j}\exp\left(\left(t_{pre}^{j}-t_{d}\right)/\tau_{2}\right), \end{array}$$ (29)

$$\begin{array}{ccc} b & = & w_{j}\exp\left(\left(t_{pre}^{j}-t_{d}\right)/\tau_{1}\right), \end{array}$$ (30)

$$\begin{array}{ccc} c & = & w_{j}\exp\left(\left(t_{pre}^{j}-t_{d}\right)/\tau_{2}\right)-w_{j}\exp\left(\left(t_{pre}^{j}-t_{d}\right)/\tau_{1}\right)-\Delta u_{j}, \end{array}$$ (31)

*the voltage variation* Δ*u*_*j*_ *of the postsynaptic neuron can be achieved by the presynaptic spike time jitter*

$$\begin{array}{c} \Delta t_{pre}^{j}=\tau_{1}\ln\left(\frac{-b\pm \sqrt{b^{2}-4ac}}{2a}\right). \end{array}$$ (32)

***Proof***: Supposing that *u*_*j*_ is the postsynaptic voltage stimulated by the input spike $t_{pre}^{j}$ at the target time *t*_*d*_, and the presynaptic spike jitter $△t_{pre}^{j}$ makes the voltage change △*u*_*j*_. By Eq (1), we have

$$\begin{array}{c} w_{j}\left(\exp\left(\frac{t_{pre}^{j}+\Delta t_{pre}^{j}-t_{d}}{\tau_{1}}\right)-\exp\left(\frac{t_{pre}^{j}+\Delta t_{pre}^{j}-t_{d}}{\tau_{2}}\right)\right)=u_{j}+\Delta u_{j}, \end{array}$$ (33)

and then

$$\begin{array}{ccc} \Delta u_{j} & = & w_{j}[\exp\left(\frac{t_{pre}^{j}-t_{d}}{\tau_{1}}\right)\exp\left(\frac{\Delta t_{pre}^{j}}{\tau_{1}}\right)-\exp\left(\frac{t_{pre}^{j}-t_{d}}{\tau_{1}}\right)\\ & & +\;\exp\left(\frac{t_{pre}^{j}-t_{d}}{\tau_{2}}\right)-\exp\left(\frac{t_{pre}^{j}-t_{d}}{\tau_{2}}\right)\exp\left(\frac{\Delta t_{pre}^{j}}{\tau_{2}}\right)]. \end{array}$$ (34)

Let

$$\begin{array}{c} z=\exp(\Delta t_{pre}^{j}/\tau_{1}), \end{array}$$ (35)

and by Eqs (29)–(31) and (34) can be expressed by

$$\begin{array}{c} az^{2}+bz+c=0. \end{array}$$ (36)

Under the condition (*I*), we have *w*_*j*_ ≠ 0 ⇒ *a* ≠ 0, and if *w*_*j*_ > 0, for condition (*II*),

$$\begin{array}{c} \Delta u_{j}\leq \left(\frac{1}{4}-\epsilon_{j}\left(t_{d}-t_{pre}^{j}\right)\right)w_{j}, \end{array}$$ (37)

$$\begin{array}{c} \epsilon_{j}\left(t_{d}-t_{pre}^{j}\right)+\frac{\Delta u_{j}}{w_{j}}\leq \frac{1}{4}. \end{array}$$ (38)

Then the discriminant of Eq (36) is

$$\begin{array}{ccc} \Delta =b^{2}-4ac & = & w_{j}^{2}\exp^{2}\left(\frac{t_{pre}^{j}-t_{d}}{\tau_{1}}\right)-4w_{j}^{2}\exp\left(\frac{t_{pre}^{j}-t_{d}}{\tau_{2}}\right)\left[\epsilon_{j}\left(t_{d}-t_{pre}^{j}\right)+\frac{\Delta u_{j}}{w_{j}}\right]\\ & \geq & w_{j}^{2}\exp^{2}\left(\frac{t_{pre}^{j}-t_{d}}{\tau_{1}}\right)-w_{j}^{2}\exp\left(\frac{t_{pre}^{j}-t_{d}}{\tau_{2}}\right)\\ & = & w_{j}^{2}\exp\left(\frac{t_{}^{j}-t_{d}}{\tau_{2}}\right)-w_{j}^{2}\exp\left(\frac{t_{pre}^{j}-t_{d}}{\tau_{2}}\right)=0. \end{array}$$ (39)

Analogously, when *w*_*j*_ < 0, for condition (*III*),

$$\begin{array}{c} \Delta u_{j}\geq \left(\frac{1}{4}-\epsilon_{j}\left(t_{d}-t_{pre}^{j}\right)\right)w_{j}, \end{array}$$ (40)

$$\begin{array}{c} \epsilon_{j}\left(t_{d}-t_{pre}^{j}\right)+\frac{\Delta u_{j}}{w_{j}}\leq \frac{1}{4}, \end{array}$$ (41)

$$\begin{array}{c} \Delta =b^{2}-4ac\geq 0. \end{array}$$ (42)

Then, under these conditions, Eq (36) has solutions

$$\begin{array}{c} z=\frac{-b\pm \sqrt{b^{2}-4ac}}{2a}. \end{array}$$ (43)

By the property of the logarithmic function, the spike jitter $\Delta t_{pre}^{j}$ can be obtained by Eq (35) only if *z* > 0. For *w*_*j*_ > 0, we have *a* < 0, *b* > 0, then

$$\begin{array}{c} z=\frac{-b-\sqrt{b^{2}-4ac}}{2a}>0. \end{array}$$ (44)

Under condition (*II*), for *w*_*j*_ > 0, $\Delta u_{j}>-w_{j}\epsilon_{j}\left(t_{d}-t_{pre}^{j}\right)$, we have $\epsilon_{j}\left(t_{d}-t_{pre}^{j}\right)+\Delta u_{j}/w_{j}>0$, and then

$$\begin{array}{c} 4ac=4w_{j}^{2}\exp\left(\frac{t_{pre}^{j}-t_{d}}{\tau_{2}}\right)\left[\epsilon_{j}\left(t_{d}-t_{pre}^{j}\right)+\frac{\Delta u_{j}}{w_{j}}\right]>0, \end{array}$$ (45)

$$\begin{array}{c} b^{2}-4ac<b^{2}, \end{array}$$ (46)

$$\begin{array}{c} z=\frac{-b+\sqrt{b^{2}-4ac}}{2a}>0. \end{array}$$ (47)

Analogously, by condition (*III*), *w*_*j*_ < 0,

$$\begin{array}{c} z=\frac{-b\pm \sqrt{b^{2}-4ac}}{2a}>0. \end{array}$$ (48)

Then, under these conditions, $\Delta t_{pre}^{j}$ is solved by Eqs (35) and (43) with

$$\begin{array}{c} \Delta t_{pre}^{j}=\tau_{1}\ln\left(\frac{-b\pm \sqrt{b^{2}-4ac}}{2a}\right). \end{array}$$ (49)

The result follows.

Specially, when Δ*u*_*j*_ exceeds the boundary of Theorem 2 (*II*) or (*III*) in our algorithm, it is set to the corresponding feasible boundary in the same direction of the condition.

### Convergence Analysis

In this section, the convergence of our algorithm is investigated. To guarantee the convergence of the traditional and our algorithms employing the SRM_0_ model, some conditions need to be met to select target time points. These conditions for traditional algorithms and our algorithm are studied in Theorem 3 (1) and Theorem 3 (2) respectively by analyzing the voltage function and the spiking firing conditions.

**Theorem 3** *In the network under* *n* *layers employing the* *SRM*_0_ *model described in* *Eqs* (1)–(3) *with* *τ*_1_ = 2*τ*_2_, *we have*:

*(1) To guarantee the convergence of the traditional algorithms based on the precise spike time mechanism, a time point* $t_{d}^{m}$ *is available as target time only if there exist input spikes in* $\left[t_{d}^{m}-\left(n-1\right)\tau_{1}\ln2,t_{d}^{m}\right)$.

*(2) To guarantee the convergence of our algorithm, a time points* $t_{d}^{m}$ *is available as target time only if there exist input spikes in* $\left[0,t_{d}^{m}\right)$. *When the strategy of* [*t*_1_, *t*_2_] *described in* Fig 2 *is applied to our algorithm, this scope is* $\left[t_{d}^{m}-nt_{1},t_{d}^{m}-nt_{2}\right]$.

***Proof***: (1) For an arbitrary *j*th input spike $t_{in}^{j}$, by Eq (2),

$$\begin{array}{ccc} \epsilon_{pre}\left(t-t_{in}^{j}\right) & = & \exp\left(-\frac{t-t_{in}^{j}}{\tau_{1}}\right)-\exp\left(-\frac{t-t_{in}^{j}}{\tau_{2}}\right)\\ & = & \exp\left(\frac{t_{in}^{j}}{\tau_{1}}\right)\exp\left(-\frac{t}{\tau_{1}}\right)-\exp\left(\frac{t_{in}^{j}}{\tau_{2}}\right)\exp\left(-\frac{t}{\tau_{2}}\right). \end{array}$$ (50)

Taking the partial derivatives,

$$\begin{array}{c} \frac{\partial \epsilon_{pre}\left(t-t_{in}^{j}\right)}{\partial t}=-\frac{1}{\tau_{1}}\exp\left(\frac{t_{in}^{j}}{\tau_{1}}\right)\exp\left(-\frac{t}{\tau_{1}}\right)+\frac{1}{\tau_{2}}\exp\left(\frac{t_{in}^{j}}{\tau_{2}}\right)\exp\left(-\frac{t}{\tau_{2}}\right). \end{array}$$ (51)

In the traditional precise time mechanism, it is $\frac{\partial \epsilon_{pre}\left(t-t_{in}^{j}\right)}{\partial t}\geq 0$ when emitting spikes, then we have

$$\begin{array}{c} -\frac{1}{\tau_{1}}\exp\left(\frac{t_{in}^{j}}{\tau_{1}}\right)\exp\left(-\frac{t}{\tau_{1}}\right)+\frac{1}{\tau_{2}}\exp\left(\frac{t_{in}^{j}}{\tau_{2}}\right)\exp\left(-\frac{t}{\tau_{2}}\right)\geq 0, \end{array}$$ (52)

$$\begin{array}{c} \exp\left(\frac{t}{\tau_{1}}-\frac{t}{\tau_{2}}\right)\geq \frac{\tau_{2}}{\tau_{1}}\left(\frac{t_{in}^{j}}{\tau_{1}}-\frac{t_{in}^{j}}{\tau_{2}}\right), \end{array}$$ (53)

by *τ*_1_ = 2*τ*_2_,

$$\begin{array}{c} \left(\frac{\tau_{2}-\tau_{1}}{\tau_{1}\tau_{2}}\right)t\geq -\ln2+\left(\frac{\tau_{2}-\tau_{1}}{\tau_{1}\tau_{2}}\right)t_{in}^{j}, \end{array}$$ (54)

$$\begin{array}{c} t\leq t_{in}^{j}+\tau_{1}\ln2. \end{array}$$ (55)

Then in traditional algorithms, the input spike $t_{in}^{j}$ can only inspire output spikes in the scope $\left(t_{in}^{j},t_{in}^{j}+\tau_{1}\ln2\right]$ for its postsynaptic neurons. Analogously, the output spikes caused by $t_{in}^{j}$ are in $\left(t_{in}^{j},t_{in}^{j}+\left(n-1\right)\tau_{1}\ln2\right]$ after *n* layers. Consequently, traditional algorithms cannot get convergent at $t_{d}^{m}$ if there is no input spike in $\left[t_{d}^{m}-\left(n-1\right)\tau_{1}\ln2,t_{d}^{m}\right)$. Then, to guarantee the convergence of the traditional algorithms based on the precise spike time mechanism, a time point $t_{d}^{m}$ is available as target time only if there exist input spikes in $\left[t_{d}^{m}-\left(n-1\right)\tau_{1}\ln2,t_{d}^{m}\right)$.

(2) Our algorithm employs the primate selective attention mechanism instead of the precise spike time rule, then there is no requirement of $\partial \epsilon_{pre}\left(t-t_{in}^{j}\right)/\partial t\geq 0$. When the local influence shown in Fig 2 is not applied to our algorithm, all presynaptic spikes which have an influence on $t_{d}^{m}$ can be trained to complete learning. Consequently, the time scope for input spikes is $\left[0,t_{d}^{m}\right)$.

**Fig 3:**
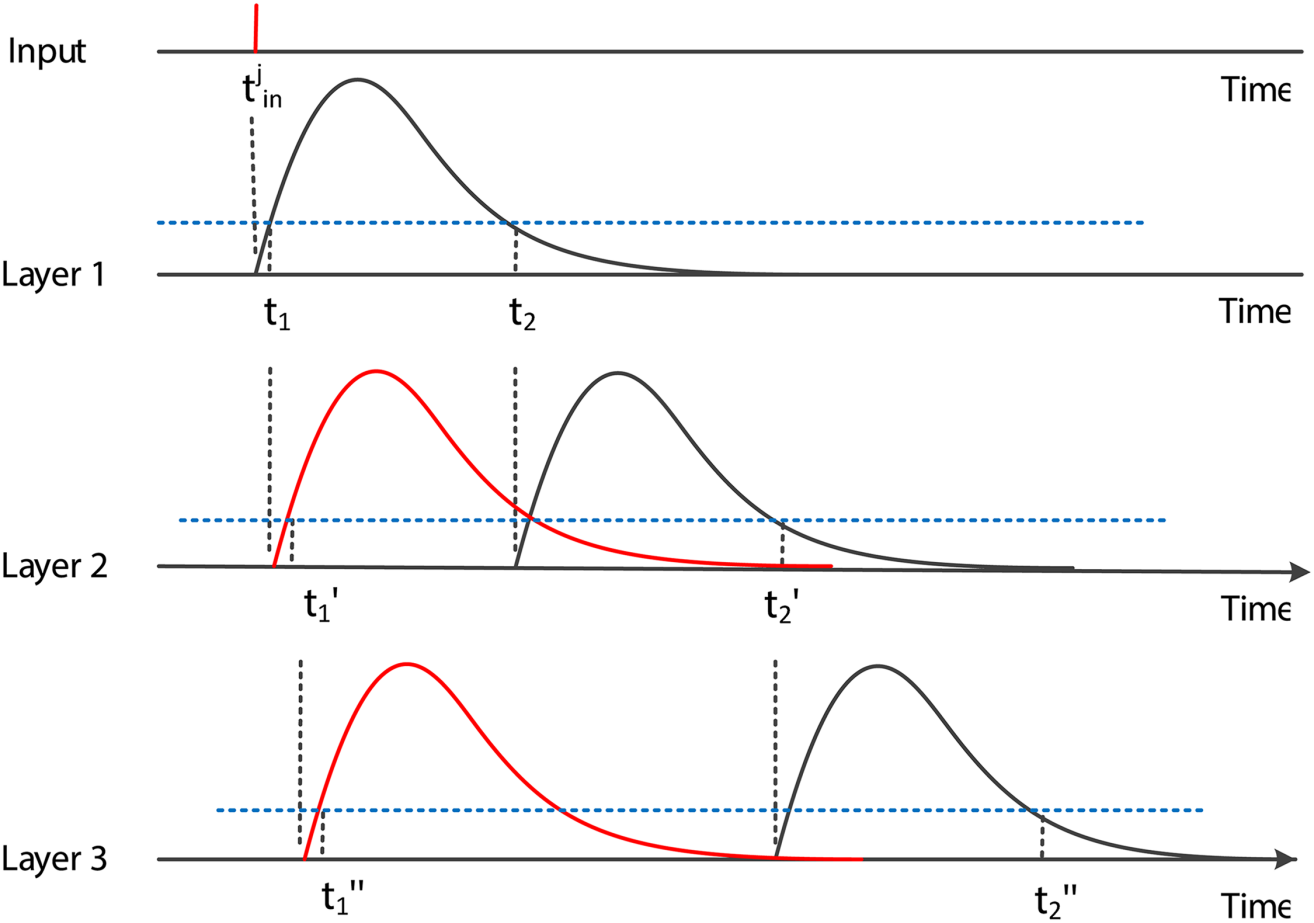
The output spike scopes. The output spike scopes generated by the input spike $t_{in}^{j}$ to each layer.

If the local influence shown in Fig 2 is applied to our algorithm, the time scope of output spikes generated by the input $t_{in}^{j}$ after several layers is shown in Fig 3. It is obvious that in layer 1, the time scope is [*t*_1_, *t*_2_], and in layer 2, the earliest time to fire is *t*_1_′ = *t*_1_ + *t*_1_, and the latest firing time is *t*_2_′ = *t*_2_ + *t*_2_. Then, for layer *n* − 1, we have *t*_*d*_ satisfying Eq (56) to complete training:

$$\begin{array}{c} \left(n-1\right)t_{1}<t_{d}^{m}<\left(n-1\right)t_{2} \end{array}$$ (56)

Obviously, if there is no input in $\left[t_{d}^{m}-nt_{1},t_{d}^{m}-nt_{2}\right]$, this $t_{d}^{m}$ can not be trained convergently. Then, in this condition, the time points $t_{d}^{m}$ is available as target time only if there exist input spikes in $\left[t_{d}^{m}-nt_{1},t_{d}^{m}-nt_{2}\right]$.

The results follow.

Theorem 3 provides conditions for encoding target times, which guarantees the convergence of different algorithms. It also indicates that the convergent condition of our algorithm is relaxed compared with traditional algorithms. When target spikes are selected following these conditions, different algorithms have different convergence properties and speed, which depend on their training mechanisms. In our algorithm, all qualified target times can be trained successfully and efficiently.

At the *m*th target time $t_{d}^{m}$, the convergence of our algorithm is proved in the Theorem 4 by analyzing the postsynaptic voltage. Since the interference from training other target spikes or patterns varies with different network status, the following theorem proves the convergence of our algorithm ignoring this interference. The convergent situations with various interference are investigated in the following simulations sections.

**Theorem 4** *In the* *SRM*_0_ *model described in* *Eqs* (1)–(3) *with* *τ*_1_ = 2*τ*_2_, *the postsynaptic voltage of our algorithm at an arbitrary target time* $t_{d}^{m}$ *is convergent to the threshold* *ϑ* *if the condition in Theorem 3 (2) holds, ignoring the interference from training other target spikes or patterns*.

***Proof***: There are two cases in the training: (1) the presynaptic hidden neurons emit qualified spikes.

Since our algorithm are trained layer-wisely, each layer shares the same training process and becomes convergent in the same way. Then, the convergence of our algorithm is proved in one layer with a postsynaptic neuron and several presynaptic neurons. Suppose that the voltage of the postsynaptic neuron at $t_{d}^{m}$ is $u(t_{d}^{m})$ calculated by Eq (1), and the voltage variations generated by the presynaptic weight modification and spike jitter are Δ*u*_*w*_ and Δ*u*_*t*_ respectively. For an arbitrary postsynaptic neuron *o*, and to the *p*th presynaptic spike, the voltage variation generated by the weight modification of this spike is $\Delta u_{w}^{p}$, which is calculated by

$$\begin{array}{c} \Delta u_{w}^{p}=\frac{\partial u(t_{d}^{m})}{\partial w_{p}}\Delta w_{p}, \end{array}$$ (57)

where Δ*w*_*p*_ is the variation of its weight *w*_*p*_. Since $\partial u(t_{d}^{m})/\partial w_{p}=\epsilon_{p}\left(t_{d}^{m}-t_{in}^{p}\right)$,

$$\begin{array}{c} \Delta u_{w}^{p}=\epsilon_{p}\left(t_{d}^{m}-t_{in}^{p}\right)\Delta w_{p}. \end{array}$$ (58)

For all presynaptic inputs, the voltage variation generated by the weight modification is

$$\begin{array}{c} \Delta u_{w}=\sum_{p=m_{1}}^{m_{2}}\epsilon_{p}\left(t_{d}^{m}-t_{pre}^{p}\right)\Delta w_{p} \end{array}$$ (59)

in which $t_{pre}^{p}$ is the *p*th presynaptic spike time, and *m*_1_ and *m*_2_ are the first and last indexes of these presynaptic spikes. By Eqs (5), (6) and (14),

$$\begin{array}{c} \Delta u_{w}=\sum_{p=m_{1}}^{m_{2}}\epsilon_{p}\left(t_{d}^{m}-t_{pre}^{p}\right)\frac{r\gamma_{p}^{n}\left(\vartheta -u\left(t_{d}^{m}\right)\right)}{\epsilon_{p}\left(t_{d}^{m}-t_{pre}^{p}\right)}=\sum_{p=m_{1}}^{m_{2}}r\gamma_{p}^{n}\left(\vartheta -u\left(t_{d}^{m}\right)\right). \end{array}$$ (60)

By Eq (15), $\sum_{p=m_{1}}^{m_{2}}\gamma_{p}^{n}=1$, then

$$\begin{array}{c} \Delta u_{w}=r\left(\vartheta -u\left(t_{d}^{m}\right)\right). \end{array}$$ (61)

If the conditions in Theorem 2 hold, according to Eq (8), the $\Delta t_{pre}^{p}$ calculated by Eq (32) is

$$\begin{array}{c} \Delta t_{pre}^{p}=\tau_{1}\ln\left(\frac{-b\pm \sqrt{b^{2}-4ac}}{2a}\right), \end{array}$$ (62)

which makes the postsynaptic voltage variation generated by the presynaptic spike jitter $\Delta u_{t}^{p}$ become $\gamma_{t}^{pn}err_{t}^{n}$, with $err_{t}^{n}$ and $\gamma_{t}^{pn}$ defined in Eqs (7) and (9) respectively. Under these conditions, for all presynaptic spikes, the voltage variation inspired by the presynaptic spike jitter Δ*u*_*t*_ is

$$\begin{array}{c} \Delta u_{t}=\sum_{p=m_{1}}^{m_{2}}\gamma_{t}^{pn}err_{t}^{n}. \end{array}$$ (63)

By Eq (9), $\sum_{p=m_{1}}^{m_{2}}\gamma_{t}^{pn}=1$, then according to Eqs (5) and (7),

$$\begin{array}{c} \Delta u_{t}=\left(1-r\right)\left(\vartheta -u\left(t_{d}^{m}\right)\right), \end{array}$$ (64)

where *r* is a parameter defined in Eq (7). Consequently, the whole postsynaptic voltage variation Δ*u* generated by both presynaptic spike jitter and weight modification is

$$\begin{array}{ccc} \Delta u & = & \Delta u_{t}+\Delta u_{m}=\left(1-r\right)\left(\vartheta -u\left(t_{d}^{m}\right)\right)+r\left(\vartheta -u\left(t_{d}^{m}\right)\right)\\ & = & \vartheta -u(t_{d}^{m}), \end{array}$$ (65)

and then

$$\begin{array}{c} u(t_{d}^{m})+\Delta u=\vartheta . \end{array}$$ (66)

If the value of $\Delta u_{t}^{p}$ exceeds the boundary in Theorem 2, the corresponding feasible boundary value is set to $\Delta u_{t}^{p}$, and the solution is obtained according to Eq (32). It is obvious that the boundary value has the same training direction with Δ*u*, which can make *err* close to 0. Then our algorithm will be convergent after several leaning epochs at $t_{d}^{m}$.

(2) If there is no qualified input spike in the presynaptic hidden layer, our algorithm adds spikes randomly with probability calculated by Eq (16), after which all weight modifications and spike jitters are the same as case (1), and our algorithm can get convergence.

The layer-wise training is employed in our algorithm, by which each layer shares the same training process and becomes convergent in the same way. In this analysis, the interference of other target spike trains or patterns are ignored. With the influence, our algorithm requires several more epochs to offset this interference and complete training.

The results follow.

### Computational Complexity

In this section, the computational time complexity of our algorithm is studied and compared with two traditional algorithms, the SpikeProp [28] and Multi-ReSuMe [32]. Before this, the detailed pseudo-codes of the feedforward and feedback processes of our method are listed.

#### Feedforward calculation

According to the previous description, the pseudo-code of the feedforward calculation of our method is listed below. Since in the multilayer networks, each layer shares the same feedforward calculation process, only the calculation of one layer is described in this pseudo-code.

**The Feedforward Calculation of Our Algorithm**

**Definition**:

*T*_*pre*_: the set of presynaptic spikes, which contains spikes emitted by all presynaptic neurons $\left\{t_{pre}^{1},t_{pre}^{2},t_{pre}^{3},\ldots ,t_{pre}^{P}\right\}$. *T*_*pre*_ is sorted and has no duplicate numbers.

**Initialization**:

The weight matrix *W* is initialized randomly.

**Feedforward calculation**:

 **For** each postsynaptic neuron:

  **For** each presynaptic spike interval $t_{pre}^{j}$ to $t_{pre}^{j+1}$ with 1 < *j* < *P* − 1:

   For all presynaptic spikes before $t_{pre}^{j}$, calculate parameters *a* and *b* by Eqs (17) and (18).

   **If** *a* and *b* meet the conditions in Theorem 1:

    Calculate the output spike time in this scope by Eq (19) and add it to the output spike train of the postsynaptic neuron.

   **End If**

  **End For**

 **End For**

Supposing that there are *M* presynaptic neurons, *N* postsynaptic neurons, *P* input spikes of all these presynaptic neurons, and the time length is *T*. As described in the pseudo-code, our algorithm detects each input spike scope and calculates parameters *a* and *b* by all of these *P* input spikes in the worst case, then the time complexity of our algorithm for one layer is *O*(*NP*^2^), which also reveals the number of operations in our method.

For traditional feedforward calculation method, all discrete time points in *T* are detected instead of the input spike scopes of *P*, then the second loop in the pseudo-code above is replaced by the time scope in *T* (supposing that the time interval is 1ms). For each time scope, it calculates the postsynaptic voltage by these *P* input spikes and determines whether the voltage is greater than the threshold. In this way, the time complexity of the traditional method in one layer is *O*(*NTP*). Since a neuron can only emits one spike in a time points, we have *P* ≤ *T*. Consequently, the time complexity of our method in the feedforward calculation is less than that of the traditional approach.

#### Feedback modification

Similar to the feedforward calculation, the feedback weight modifications in each layer and each output neuron of our algorithm have the same training process. Then in this part, only the training process of one layer and one output neuron is listed in the following pseudo-code.

**The Feedback Modification of Our Algorithm**

**Definition**:

*T*_*pre*_: the set of presynaptic spikes, which contains spikes emitted by all presynaptic neurons $\left\{t_{pre}^{1},t_{pre}^{2},t_{pre}^{3},\ldots ,t_{pre}^{P}\right\}$.

*T*_*d*_: the set of target output spikes, which contains all target spikes of the postsynaptic neuron $\left\{t_{d}^{1},t_{d}^{2},t_{d}^{3},\ldots ,t_{d}^{D_{1}}\right\}$.

**Initialization**:

The weight matrix *W* is initialized randomly.

**Feedback modification**:

 **For** each target spike time in *T*_*d*_:

  Calculate the weighted sum of all input spikes as the postsynaptic voltage *u*(*t*_*d*_), and calculate the error *err* by Eq (5).

  **If** *err* ≠ 0

   **Step1**: Assign *err* to each layer by Eqs (6) and (7).

   **Step2**: Calculate the presynaptic spike variation in the current layer by Eq (10).

   **Step3**: Adjust all presynaptic weights in this layer by Eq (14).

  **End If**

 **End For**

Assuming that there are *M* presynaptic neurons, *N* postsynaptic neurons, and the number of target spikes for all postsynaptic neurons is *D*, which is equal to *D*_1_ + *D*_2_ + … + *D*_*N*_, with *D*_*i*_ represents the number of target spikes of the *i*th postsynaptic neuron. The number of input spikes is *P*, and the time length is *T*. According to the pseudo-code for the feedback modification of our algorithm, the time complexity for one layer is *O*(*DP*), which also reveals the number of operations in our algorithm.

In traditional algorithms, like the SpikeProp and Multi-ReSuMe, the postsynaptic states at all time points of *T* instead of target intervals *T*_*d*_ are detected and their corresponding weights are modified by a given condition. Then the time complexity for most traditional algorithms like the SpikeProp and Mullti-ReSuMe in one layer is *O*(*TP*). Since *D* < *T*, the number of operations in our algorithm is less than traditional methods.

## Training Performance

In this section, the training performance of our algorithm is investigated and compared with two classical algorithms, the SpikeProp [28] and Multi-ReSuMe [32].

**Fig 4:**
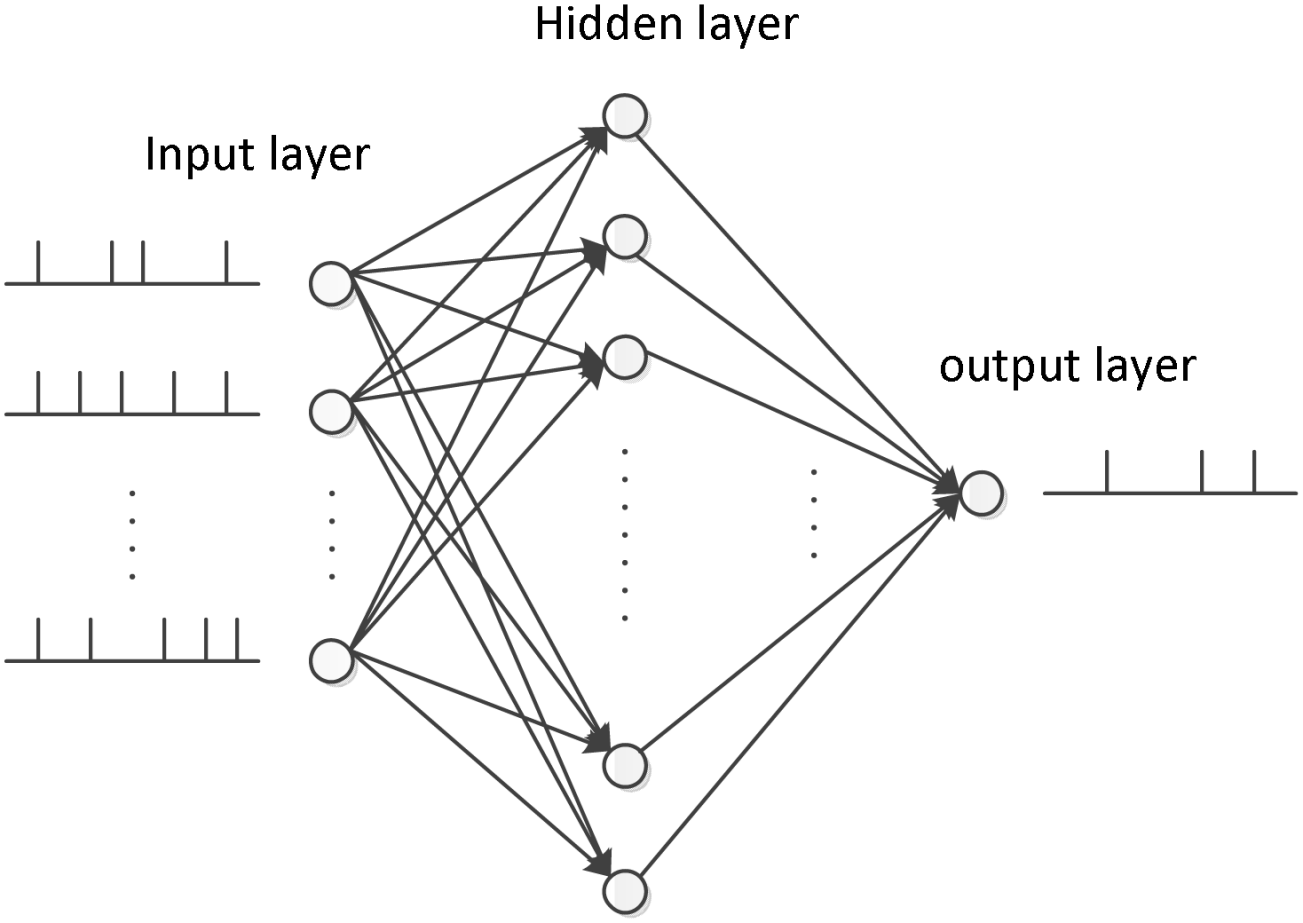
The network structure in our simulation. There are 50 input neurons, 100 hidden neurons, and one output neuron.

A spiking network structure employing an input layer with 50 neurons, a hidden layer with 100 spiking neurons, and an output neuron is devised in our simulations, which is shown in Fig 4. The training of the multilayer neural network consists of two steps, the feedforward calculation and feedback weight modification. The efficiency of the both two steps is studied in the following parts.

### Feedforward Calculation

The feedforward calculation is an important step before learning, it computes the output spikes from the input ones. In this subsection, two simulations are conducted to investigate the computational performance of our proposed method described in Theorem 1 compared with the traditional precise time calculation method. Specifically, the network structure is shown in Fig 4, and these input output spikes are generated by a homogeneous Poisson process.

The first simulation is carried out to explore the feedforward calculation performance in different time lengths from 200 ms to 2800 ms, and for each input neuron, one spike generated by a homogeneous Poisson process is emitted. To evaluate the similarity of the output spike trains calculated by our algorithm and traditional method quantitatively, the correlation-based measure *C* [38] is employed, with

$$\begin{array}{c} C=\frac{v_{1}\cdot v_{2}}{|v_{1}\left|\right|v_{2}|}, \end{array}$$

where ***v***_**1**_ **⋅** ***v***_**2**_ is the inner product, and |***v***_**1**_|, |***v***_**2**_| are the Euclidean norms of ***v***_**1**_ and ***v***_**2**_ respectively. The ***v***_**1**_ and ***v***_**2**_ are vectors obtained by the convolution of the two spike trains using a Gaussian filter:

$$\begin{array}{c} v_{i}\left(t\right)=\sum_{m=1}^{N_{i}}\exp[-{\left(t-t_{m}^{i}\right)}^{2}/\sigma^{2}], \end{array}$$

where *N*_*i*_ is the number of spikes in the test spike train, and $t_{m}^{i}$ is the *m*th spike in it. *σ* is the standard deviation of this Gaussian filter which is set to be 1 in our study. Generally, the measure *C* equals to 1 for identical spike trains and decrease towards zero for loosely correlated spike trains.

**Fig 5:**
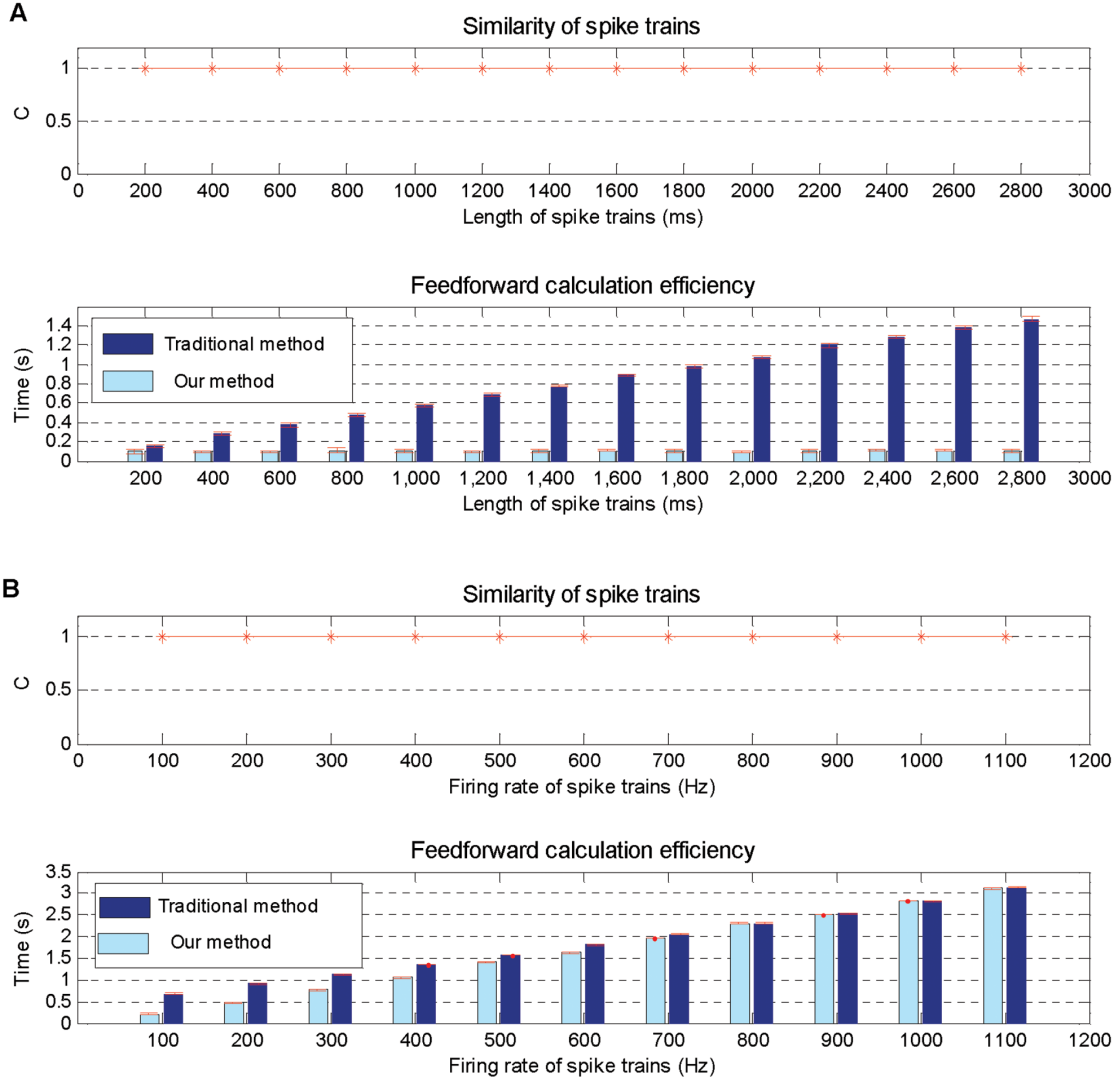
Feedforward calculation performance on various situations. A: Simulation results on different time lengths ranging from 200 ms to 2800 ms. B: Simulation results on different input firing rates ranging from 10 Hz to 1100 Hz.

The simulation results are shown in Fig 5A, which indicate that our proposed method has the same output spike trains as the traditional method, but achieves higher efficiency than it, because our method detects only time intervals of the input spikes instead of all time points in the traditional method.

In the second simulation, the performance of our proposed method is tested with the input firing rate of a homogeneous Poisson process ranging from 100 Hz to 1100 Hz with time length fixed to 1000 ms. Obviously, the higher the input firing rate, the higher the input densities, and when the firing rate is higher than 1000 Hz, the density of input spikes and all time points is similar.

Simulation results shown in Fig 5B indicate that our method has the same output spike trains as the traditional ones, and its computational time is growing with the increase of the input spike density. However, our method is still a little more efficient than the traditional method even if the input spike density is similar to that of all time intervals with a rate above 1000 Hz.

### Feedback Weight Modification

In this section, the training performance of our algorithm is investigated and compared with the traditional classical multi-layer algorithms, the Spikeprop and Multi-ReSuMe. The first simulation is devised to study the learning efficiency at different time lengths of spike trains, in which there are 50 input neurons, 100 hidden neurons, and one output neuron with a network structure depicted in Fig 4. The input spike train of each input neuron is generated by a homogeneous Poisson process with *r* = 10 Hz, ranging the time length from 200 ms to 2800 ms. The output neuron is desired to emit only one spike in these time lengths because the SpikeProp cannot complete training for multiple target spikes. For fast convergence of traditional algorithms, the target time *t*_*d*_ is set to *t*_*o*_ + 5, where *t*_*o*_ is the output firing time of the first epoch. Similar to the previous simulation, *C* is employed here to measure the accuracy.

**Fig 6:**
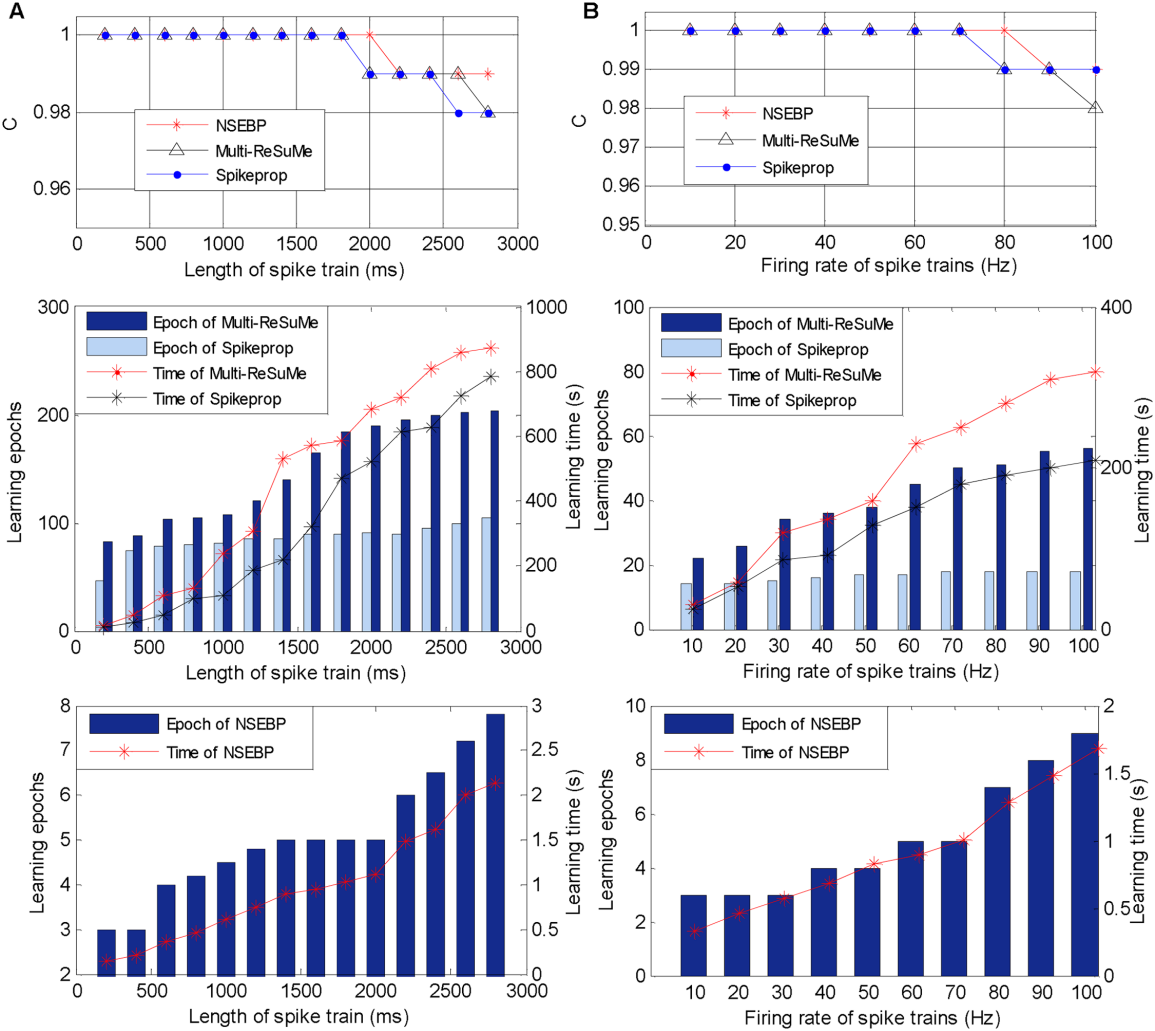
Training performance on various situations. A: Simulation results on different time lengths fixing the input spike rate to 10 Hz. B: Simulation results on different input firing rates with the time length 500 ms.

The comparison results are shown in Fig 6A, in which the upper sub-figure depicts the learning accuracy of these three algorithms. It illustrates that in this simulation, our algorithm has a similar accuracy as traditional ones. The middle sub-figure of Fig 6A shows the learning efficiency of the SpikeProp and the Multi-ReSuMe, and the efficiency of our algorithm is displayed independently in the below sub-figure because the magnitude of the learning epochs and learning time of our algorithm is not the same as that of the traditional algorithms. The comparison of these two sub-figure denotes that our algorithm requires less learning epochs and learning time than the SpikeProp and Multi-ReSuMe in various situations.

The second simulation is conducted to test the learning efficiency of our algorithm under different firing rates, in which the input and output spike trains share the same time length of 500 ms, and the input spike train of each input neuron is generated by a homogeneous Poisson process ranging from 10 Hz to 100 Hz.

The simulation results are shown in Fig 6B, where the upper sub-figure shows the accuracy of these three algorithms, the middle and the below ones depict the learning efficiency of traditional algorithms and our algorithm respectively. Similar with the previous simulation, our algorithm achieves an approximate accuracy with the SpikeProp and Multi-ReSuMe, and improves the training efficiency significantly both in training epochs and training time.

To further explore the learning efficiency of these algorithms, the training time of one epoch is tested in various firing rates and time lengths. Firstly, the time length is fixed to 500 ms, and each input neuron emits a spike train generated by a homogeneous Poisson process with firing rate ranging from 10 Hz to 100 Hz, and the output neuron emits only one spike.

The simulation results shown in Table 1 indicate that the higher the firing rate, the more time required for these three algorithms to complete one training epoch, since more input spikes required to be trained. Besides, our algorithm consumes less time than traditional ones in various firing rates.

**Table 1:** Training time of one epoch for various firing rates.

Firing rate (Hz)	Time of NSEBP (s)	Time of SpikeProp (s)	Time of Multi-ReSuMe (s)
10	0.312	0.812	0.471
20	0.441	1.255	0.814
30	0.552	1.744	1.218
40	0.634	2.185	1.598
50	0.715	2.762	2.077
60	0.746	3.278	2.468
70	0.767	3.817	2.936
80	0.771	4.496	3.422
90	0.781	5.023	3.893
100	0.794	5.591	4.314

Secondly, in order to verify the effect of the time length on the training efficiency, another simulation is carried out where each input neuron emits two spikes and the output neuron emits one spike generated by a homogeneous Poisson process, and the maximum time length varies form 100 ms to 1000 ms.

The simulation results are shown in Table 2, which denotes that in various time lengths, our algorithm has the similar running time for training one epoch, while the time of the SpikeProp and Multi-ReSuMe increases obviously. This reveals the advantage of the selective attention mechanism which enables our NSEBP to concentrate attention on target contents and does not have to scan all time points as traditional algorithms do. It also indicates that the running time of NSEBP has no direct relation to the time length. These simulations in this section demonstrate that our algorithm achieves a significant improvement in efficiency compared with traditional algorithms.

**Table 2:** Training time of one epoch for various time lengths.

Time length (ms)	Time of NSEBP (s)	Time of SpikeProp (s)	Time of Multi-ReSuMe (s)
100	0.117	0.144	0.112
200	0.123	0.238	0.125
300	0.121	0.351	0.146
400	0.123	0.477	0.199
500	0.124	0.611	0.241
600	0.127	0.751	0.293
700	0.123	0.857	0.361
800	0.128	0.978	0.416
900	0.123	1.036	0.537
1000	0.128	1.297	0.595

## Non-linear Spike Pattern Classification

### The XOR Benchmark

In this section, we perform experiments with the NSEBP on a classical example of a non-linear problem, the XOR benchmark to investigate its classification capability and the influence of different parameters. The network architecture shown in Fig 4 is employed in this section, with 4 input neurons, 10 hidden neurons and one output neuron.

**Fig 7:**
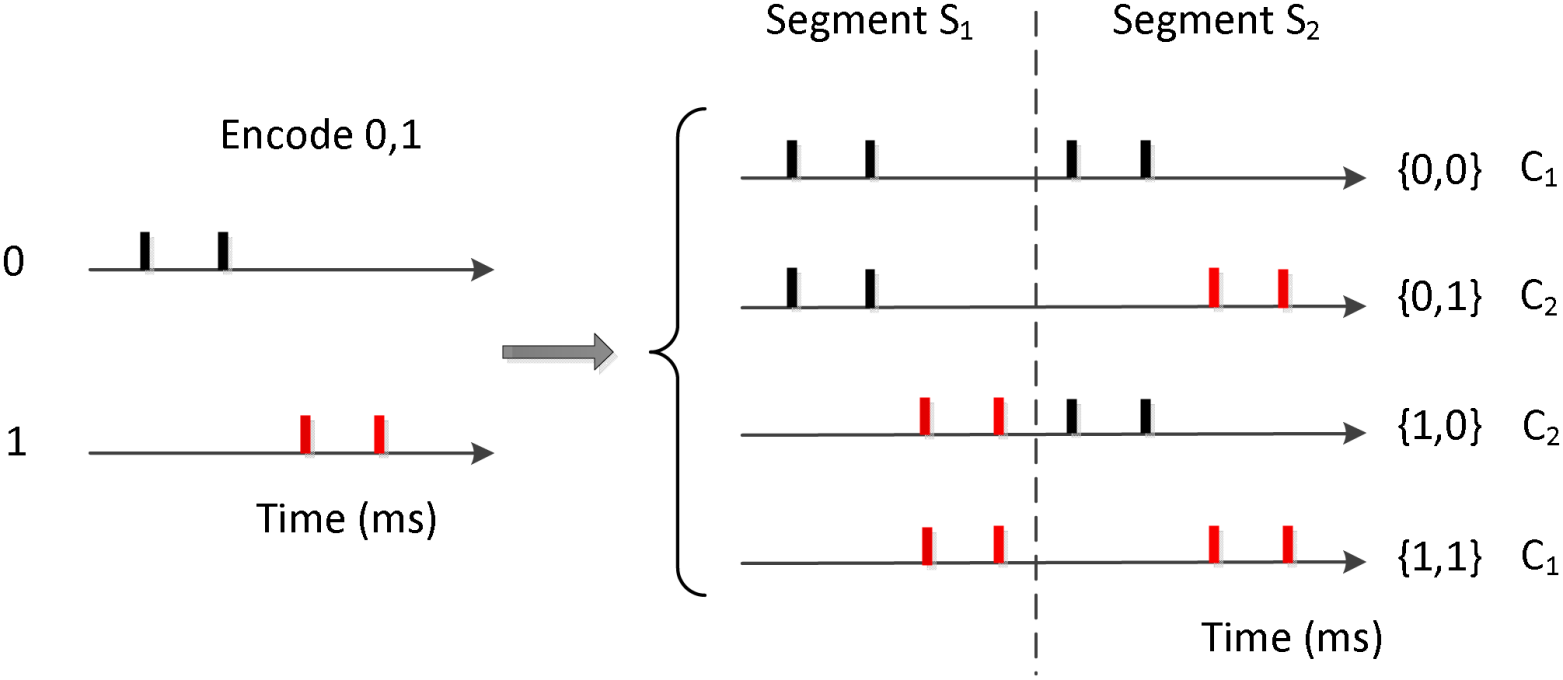
Generation of the input spike trains. Generation of the input spike trains for the classification task in the XOR problem.

The encoded method and the generation of the input spike trains are shown in Fig 7. It depicts that the input 0 and 1 are encoded randomly, which is set to the spike time [1, 2] and [3, 4] respectively in this simulation. Then the four input patterns {0, 0}, {0, 1}, {1, 0}, {1, 1} are encoded by two segments *S*_1_ and *S*_2_ copying the encoded results of 0 and 1. The classification objects are the input spike patterns, among which the input spike trains corresponding to {0, 0} and {1, 1} are one class *C*_1_, and the input spike trains corresponding to {0, 1}, {1, 0} are the other class *C*_2_. The desired outputs of the output neuron corresponding to *C*_1_ and *C*_2_ are set to 10 ms and 15 ms respectively satisfying the convergent condition in Theorem 3.

**Fig 8:**
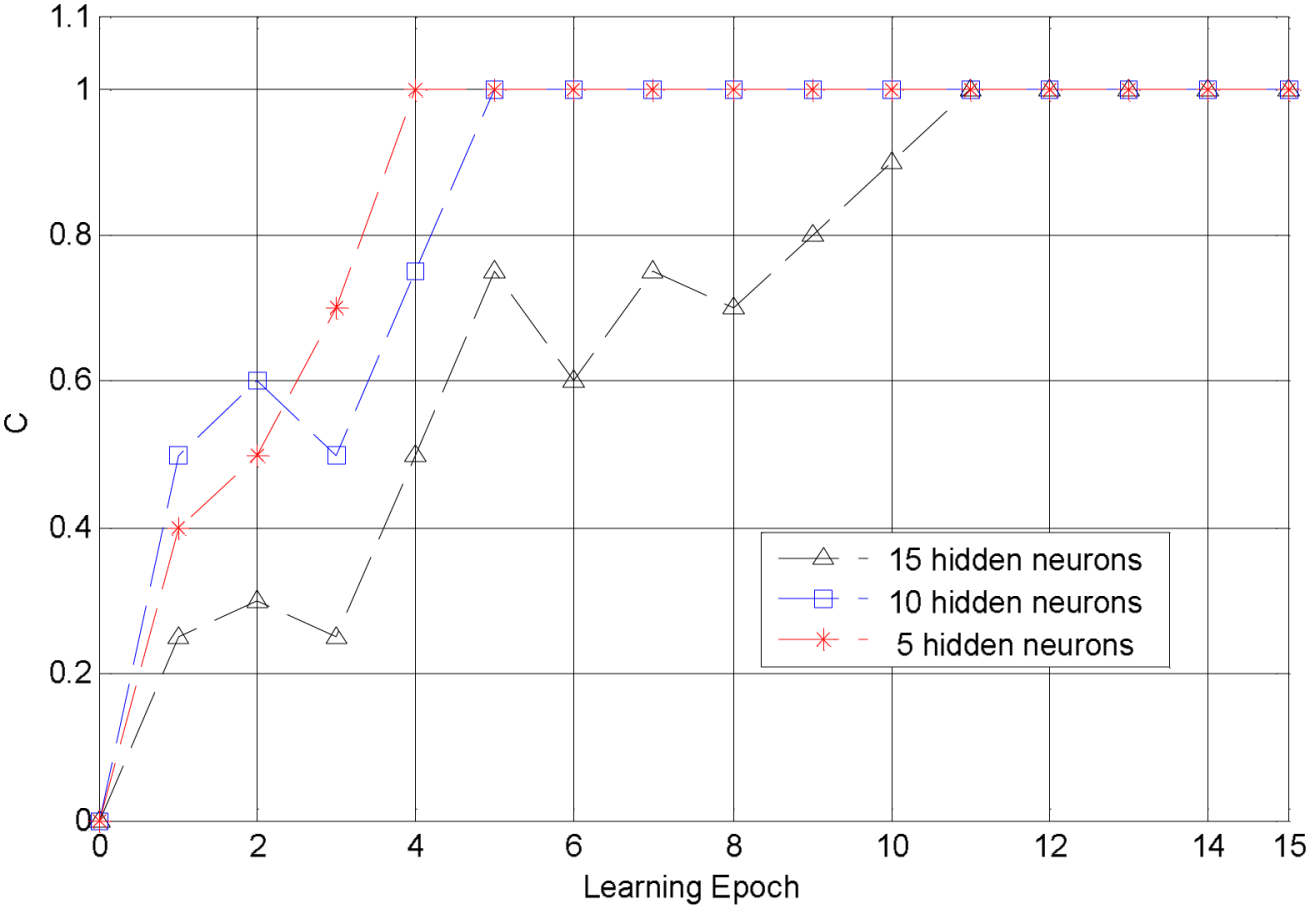
The convergent process of our algorithm. The convergent process of our algorithm with the number of hidden neurons 5, 10, 15. All of these simulations achieve accuracy 1.

Our algorithm is applied to the feed-forward network described above with *ϑ* = 1, *τ*_1_ = 5 and *r* = 0.5. Different from traditional multi-layer networks in [28, 32], our algorithm requires none sub-connections, which reduces the number of weight modification. With these parameters, our algorithm can complete training efficiently in 15 learning epochs and achieve accuracy 1 in various number of hidden neurons, as shown Fig 8. This training efficiency is higher than traditional algorithms that is at least 63 epochs in Multi-ReSuMe [32] and 250 cycles in SpikeProp [28]. In the following we systematically vary the parameters of our algorithm and investigate their influence.

### The Parameters

In this part, we explore the influence of the parameter *τ*_1_, the number of hidden neurons, the parameter *r* defined in Eq (6), and the threshold *ϑ* on the convergent epochs. 50 simulations are carried out and the average learning epoch is obtained.

**Fig 9:**
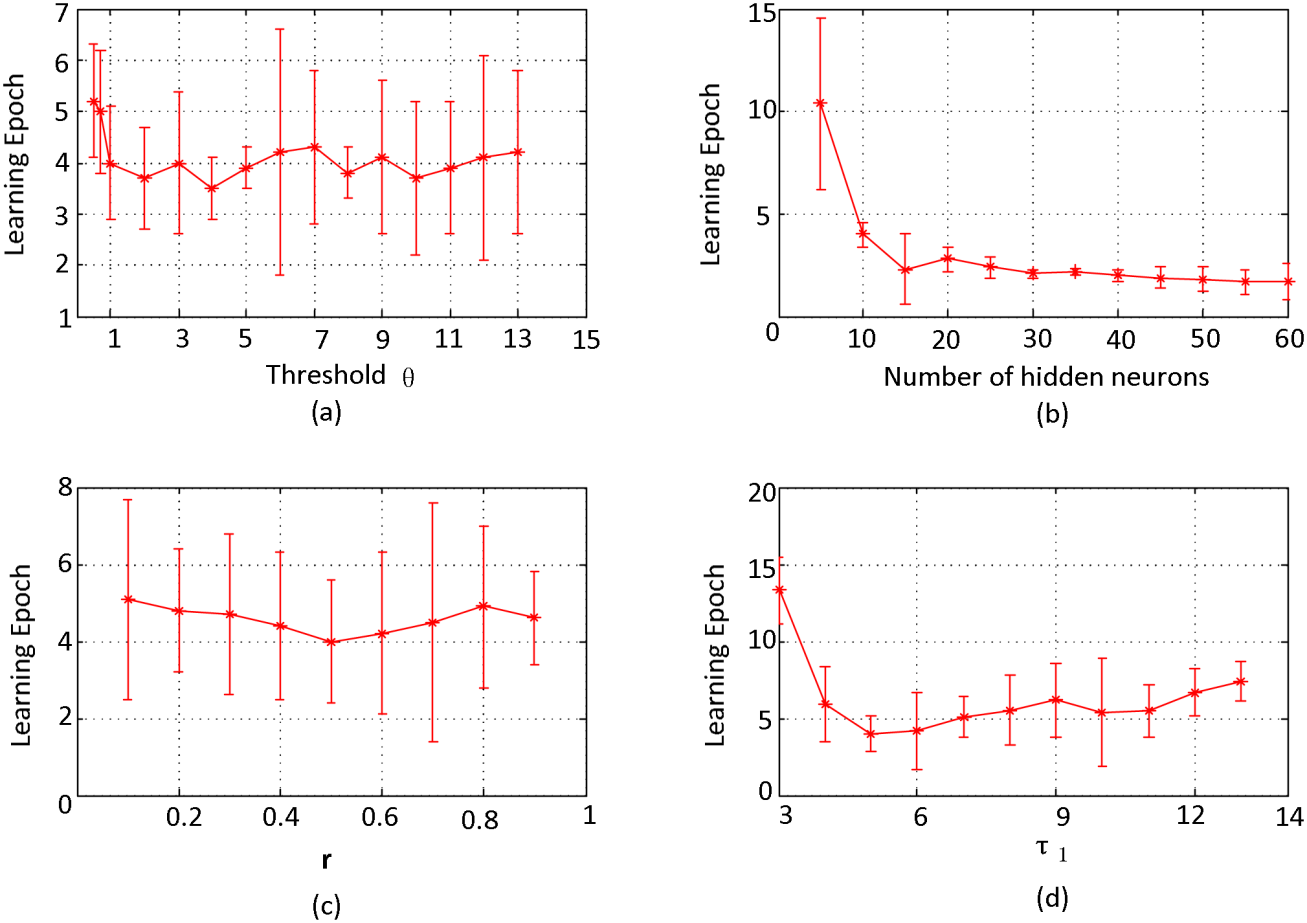
Convergent epochs with various parameters. Convergent epochs with various values of parameter *ϑ* (a), number of hidden neurons (b), r (c), and *τ*_1_ (d). All of these simulations achieve accuracy 1.

Fig 9(a) shows the convergent epochs for different values of the threshold *ϑ*, with the number of hidden neurons fixed to 10, *r* = 0.5, and *τ*_1_ = 5. It suggests that the convergence of our algorithm is insensitive to the threshold, and it can complete training in 8 epochs in various situations.

Fig 9(b) depicts the convergent epoch with different numbers of hidden neurons, which indicates that in the beginning, more neurons in the hidden layer lead to less learning epochs, while when it above 30, this change is not obvious. This is mainly because more hidden neurons make more sparse representation of the input patterns in the hidden layer, which are easier to be trained because there is less interference between different patterns. Different amount of information requires different numbers of hidden neurons for a sparse representation, and if the number of hidden neurons is large enough, the variation of the convergent epoch is not apparent.

Fig 9(c) displays the convergent epoch with different *r* defined in Eq (6), which determines the proportion of the error back-propagated to the previous layers. The simulation results demonstrate that the convergence of our algorithm has no noticeable relationship with *r*. To balance the load of each layer, we suggest *r* = 1/*n* when there are *n* layers required to be trained in our algorithm. In real world applications, *r* can be set to different values according to different requirements. Fig 9(d) shows the convergent epoch for different *τ*_1_, which indicates that our algorithm can achieve rapid convergence in various cases, and different values of *τ*_1_ have some influence on the convergent speed, but not obvious.

Simulations in this section demonstrate that our algorithm can complete non-linear classification efficiently. Besides, simulation results shown in Fig 9 indicate that our algorithm is not sensitive to various parameters, which makes our algorithm more convenient to be applied to various applications.

## Classification on the UCI Datasets

In this section, we apply NSEBP to classify both the Iris and Breast Cancer Wisconsin (BCW) datasets of the UCI [39] to investigate the capability of our algorithm over classification tasks.

### Iris Dataset

The Iris dataset is firstly applied to benchmark our algorithm. It contains three classes, each with 50 samples and refers to a type of the iris plant: Iris Setosa (class 1), Iris Versicolour (class 2), and Iris Virginica (class 3) [40]. Each sample contains four attributes: sepal length (feature 1), sepal width (feature 2), petal length (feature 3), and petal width (feature 4).

**Fig 10:**
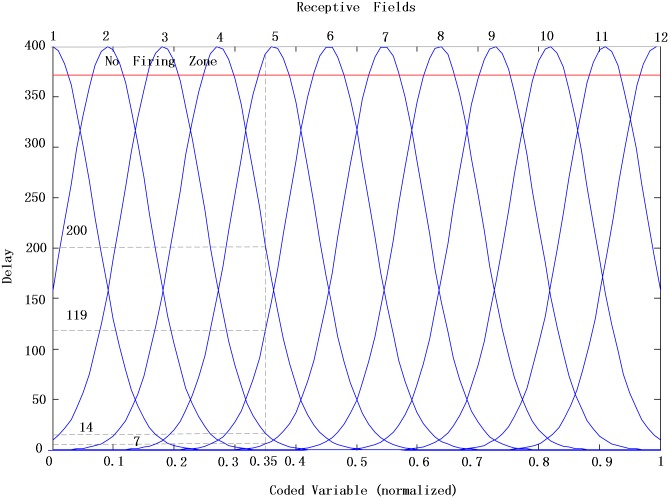
Continuous input variable encoded by means of local receptive fields. The input variable is normalized to [0, 1], and a non-firing zone is defined to avoid spikes in later time. Every no firing neuron has code −1. For instance, 0.35 is encoded to a spike train of 12 neurons: (−1, −1, 14, 200, −1, 119, 7, −1, −1, −1, −1, −1).

To make the difference between the data apparent, the data of each feature are mapped into a high dimensional space using the population time encoding method [41]. There are 12 uniforming distributed Gaussian receptive fields in [0, 1], which distribute an input variable over 12 input neurons which are shown in Fig 10.

**Fig 11:**
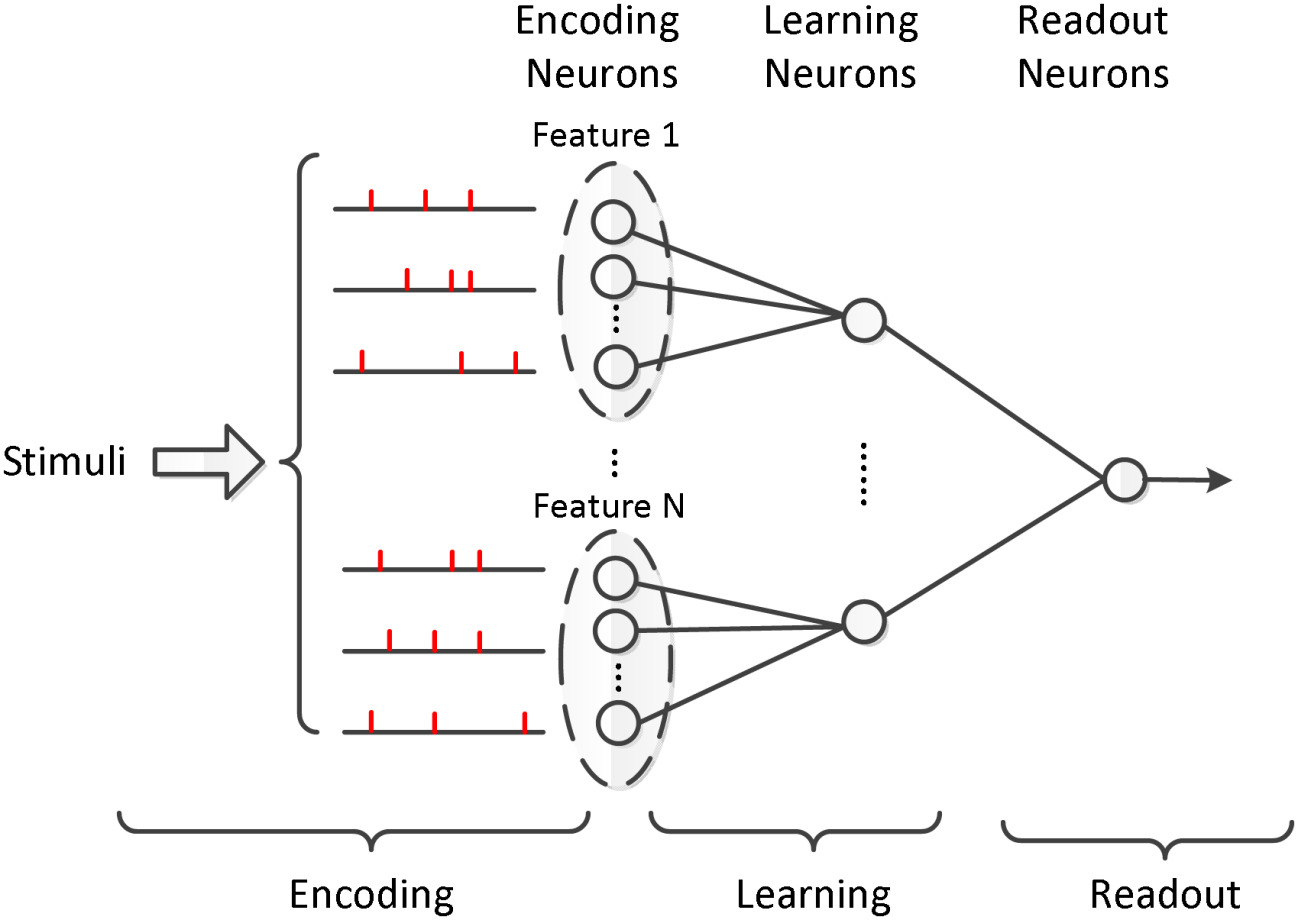
Network structure for classification. Network structure consisting of 12 ∗ *F* input neurons, *F* hidden neurons and one output neuron, where *F* is the number of features.

The network structure devised for this classification task is shown in Fig 11, in the iris data set, the input layer has 48 neurons with each feature 12 inputs. The hidden layer has 4 neurons, each possesses local connections with 12 neurons of the input layer that represent one feature. In the training period, only synaptic weights from input to hidden neurons are adjusted, and the output neuron has weight 1 which is applied to decision making.

In this application, the *i*th sample of class *c* has the input spike train ${\text{ti}}_{c}^{i}$ for four features and a target spike train ${\text{td}}_{c}^{i}$ which is obtained by ${\text{td}}_{c}^{i}={\text{ti}}_{c}^{i}+2\tau_{2\ln2}$, with parameter *τ*_2_ defined in Eq (2). The local connections in our network enable each feature to train an independent sub-network with 12 synapses. Since samples of one class have similar spike trains, encoding results reveal that there are only few different target time trains for each feature. As in the previous simulations, our algorithm chooses the class with minor voltage error.

Table 3 compares the classification accuracy and convergent epoch of the NSEBP with four classical neural network classifier: Multi-ReSuMe [32], Spikeprop [28], MatlabBP, SWAT [22] for the Iris dataset on the training set and testing set. The classifier of the Multi-ReSuMe and Spikeprop are conducted with three layer SNN structures proposed in [32] and [28] respectively. The SWAT employs a SNN architecture of 13 input neurons with each connects to 16 neurons in middle layer, and the squared cosine encoding method is employed [22]. The simulation results are shown in Table 3.

**Table 3:** Comparison Results for Iris Dataset.

Classifier	Training accuracy	Testing accuracy	Training epochs
Matlab BP	0.98	0.95	2.6 ⋅ 10^6^
SpikeProp [28]	0.97	0.96	1000
SWAT [22]	0.95	0.95	500
Multi-ReSuMe [32]	0.96	0.94	174
NSEBP	0.98	0.96	18

The comparison results indicate that our algorithm achieves comparable or even higher accuracy compared with the traditional classifiers, while our algorithm only requires 18 epochs to complete training, instead of 2.6 ⋅ 10^6^ epochs for the MatlabBP, 1000 epochs for Spikeprop, 500 epochs for the SWAT, and 174 for multi-RuSuMe. The simulation results prove that our algorithm is the most efficient one, and outperforms the compared neural networks methods significantly.

### Breast Cancer Wisconsin Dataset

The two-class Breast Cancer Wisconsin (BCW) dataset is also applied to analyze our algorithm. This dataset contains 699 samples, while 16 samples are abandoned because of missing data. Each sample has nine features obtained from a digitized image of a fine needle aspirate (FNA) of a breast mass [42].

The same network structure and training settings as these in the Iris classification simulations are employed here. There are nine features instead of four, then there are 108 input neurons and 9 hidden neurons in the network structure depicted in Fig 11.

Table 4 compares the accuracy and efficiency of the NSEBP against the existing algorithms for the BCW dataset. It shows that the test data accuracy of the NSEBP is comparable to that of the other approaches, while the NSEBP only requires 16 epochs for complete training, instead of 1500 epochs for the Spikeprop, 500 epochs for SWAT, and 9.2 ⋅ 10^6^ epochs for MatlabBP. Then, the training efficiency of our algorithm is improved significantly compared with these classical neural network algorithms.

**Table 4:** Comparison Results for BCW Dataset.

Classifier	Training accuracy	Testing accuracy	Training epochs
MatlabBP	0.98	0.96	9.2 ⋅ 10^6^
SpikeProp [28]	0.98	0.97	1500
SWAT [22]	0.96	0.96	500
NSEBP	0.97	0.96	16

Simulation results in this section demonstrate that our algorithm achieves a higher efficiency and even a higher learning accuracy than the traditional neural network methods in the classification tasks. The selective mechanism and the presynaptic spike jitter adopted in our algorithm make the efficiency of the NSEBP to be independent with the length of the spike train, and overcome the drawbacks of low efficiency in traditional back-propagation methods. With this efficiency, the training of SNNs can meet the requirement of real world applications.

## Conclusion

In this paper, an efficient multi-layer supervised learning algorithm, the NSEBP, is proposed for spiking neural networks. The accurate feedforward calculation and weight modification employing the normalized PSP learning window enables our algorithm to achieve a rapid convergence. Besides, motivated by the selective attention mechanism of the primate visual system, our algorithm only focuses on the main contents in the target spike trains and ignores neuron states at the un-target ones, which makes our algorithm to achieve a significant improvement in efficiency of training one epoch. Simulation results demonstrate that our algorithm outperforms traditional learning algorithms in learning efficiency, and is not sensitive to parameters.

The classification results on the UCI data sets indicate that the generalization ability of our algorithm is a little better than the traditional backpropagation method, and similar to the SpikeProp, but lower than the SWAT. It means that our algorithm does not make great contribution to the over fitting problem. However, the traditional methods to improve the training generalization ability can also be applied to our algorithm, such as employing an optimized network structure and better decision-making method, or better sample validate methods, that will be studied in the future work.

Our algorithm is derived from the SRM_0_ model, but the same derivation process is feasible to other models when the voltage *u* can be expressed by an equation of time *t* and can be transformed to a quadratic function by the substitute method. Besides, the proposed feed forward calculation method can be applied to the existing algorithms to improve their learning performance. Consequently, employing these training strategies, the SNNs can be applied efficiently to various applications with multilayer network structure and arbitrary real-valued analog inputs.

## Supporting Information

S1 Table Experiment Data for Feedforward Calculation Performance. (XLSX)

S2 Table Experiment Data for Training Performance with Different Time Lengths. (XLSX)

S3 Table Experiment Data for Training Performance with Different Firing Rates. (XLSX)

S4 Table Experiment Data for Training time of one epoch. (XLSX)

S5 Table Experiment Data for The Convergent Process of Our Algorithm with Different Number of Hidden Neurons. (XLSX)

S6 Table Experiment Data for The Convergent Epochs with Different Parameters. (XLSX)

S7 Table Experiment Data for Iris Dataset. (XLSX)

S8 Table Experiment Data for Breast Cancer Winsconsin Dataset. (XLSX)

S1 File Data Descriptions. (DOCX)
