# Supplementary material for: An Efficient Supervised Training Algorithm for Multilayer Spiking Neural Networks
Source: PLoS One. 2016 Apr 4;11(4):e0150329. doi: 10.1371/journal.pone.0150329 (PMC4820126; doi:10.1371/journal.pone.0150329)
Supplement: S1 File — (DOCX) [file pone.0150329.s009.docx]

**Data Descriptions:**

1. The experiment data in `S1 Table’ is for the feedforward calculation performance in the simulations of the ‘Feedforward Calculation’ Section, which is shown in Fig. 5 in our manuscript.
2. The experiment data in `S2 Table’ is for the training performance with different time lengths in the simulations of the ‘Feedback Weight Modification’ Section, which is shown in Fig. 6A in our manuscript.
3. The experiment data in `S3 Table’ is for the training performance with different firing rates in simulations of the ‘Feedback Weight Modification’ section, and shown in Fig. 6B in our manuscript.
4. The experiment data in `S4 Table’ is for the training time of one epoch in simulations of the ‘Feedback Weight Modification’ Section, which is shown in Table 1 and Table 2 in our manuscript.
5. The experiment data in `S5 Table’ is for the convergent process of our algorithm with different number of hidden neurons, which is in simulations of the ‘The XOR Benchmark’ part in the ‘Non-linear Spike Pattern Classification’ section, and shown in Fig. 8 in our manuscript.
6. The experiment data in `S6 Table’ is for the convergent epochs with different parameters, which is in simulations of the ‘The Parameters’ part in the ‘Non-linear Spike Pattern Classification’ section, and shown in Fig. 9 in our manuscript.
7. The experiment data in `S7 Table’ is for the Iris dataset in the simulations of the ‘Iris Dataset’ part in the ‘Classification on the UCI Data sets’ section, and shown in Table 3 in our manuscript.
8. The experiment data in `S8 Table’ is for the Breast Cancer Wisconsin dataset in the simulations of the ‘Breast Cancer Wisconsin Dataset’ part in the ‘Classification on the UCI Data sets’ section, and shown in Table 4 in our manuscript.
